# Emotional synchrony in family networks is linked to adolescent well-being in daily life

**DOI:** 10.1371/journal.pone.0362751

**Published:** 2026-11-11

**Authors:** Saida Heshmati, Jaymes Paolo Rombaoa, Courtney M. Allred, Alexander P. Christensen, Stacey N. Doan

**Affiliations:** 1 School of Social Science, Policy & Evaluation, Claremont Graduate University, Claremont, California, United States of America; 2 Peabody College of Education & Human Development, Vanderbilt University, Nashville, Tennessee, United States of America; 3 Department of Psychological Science, Claremont McKenna College, Claremont, California, United States of America; University of Wyoming College of Health Sciences, UNITED STATES OF AMERICA

## Abstract

Family emotional processes play a central role in adolescent well-being, yet most research relies on dyadic or static approaches that do not capture the dynamic, system-level nature of family emotional life. In a sample of adolescents (ages 12–17), mothers, and fathers (*N* = 120; 2,365 assessments), constituting 40 family triads, we used ecological momentary assessment and a dynamic network approach to estimate how emotions fluctuate over time and co-vary across family members within each system. Emotional synchrony—defined as the extent to which family members’ affective states change together—was modeled at both overall (between-family) and momentary (within-family) levels. Results indicated that families exhibited greater synchrony in positive affect than negative affect, alongside substantial heterogeneity in network structures. At the person level, greater positive affect synchrony was associated with higher adolescent well-being and lower distress, whereas negative affect synchrony showed inverse patterns. At the momentary level, increases in negative affect synchrony were associated with higher concurrent well-being and lower stress. Many of these person-level associations were substantially attenuated once adolescents’ general affect levels were accounted for, whereas synchrony at the between-family level continued to predict adolescents’ momentary well-being beyond their concurrent affect. These findings highlight emotional synchrony as a dynamic property of family systems and underscore the importance of modeling family processes across multiple timescales.

## Introduction

Families are the primary social context in which children and adolescents develop emotional competencies, regulate affect, and learn to navigate interpersonal relations. The emotional climate of families have robust associations with adolescent psychological adjustment including internalizing problems [1], emotion regulation [2–4] and well-being [5,6]. Yet, to date the majority of emotion research in families has examined these processes either within individuals or within dyads, despite the fact that the majority of children live in two-parent homes [7]. Thus, to date the research on emotional processes that fails to consider the system level of emotional family functioning is limited at best, and inaccurate worse. In the current paper, we used ecological momentary assessment (EMA; [8]) and dynamic network modeling [9] approaches to examine emotional synchrony across family triads consisting of mothers, fathers, and adolescents and its associations with adolescent well-being and distress.

Adolescence is a unique period of development characterized by significant change in both emotional reactivity and reorganization of social relationships [10], including increasing autonomy-seeking, a growing orientation toward peers, and the renegotiation of closeness with parents [11]. Because these shifts unfold within the broader family system rather than any single relationship in isolation, adolescence is a particularly informative window to examine family level emotional dynamics using a triadic approach. Importantly, families with adolescents are characterized by increased emotional variability [12–14], conditions under which system-level processes may be especially consequential for youth adjustment. In the current paper, we use ecological momentary assessment (EMA) and dynamic network modeling approaches to examine emotional synchrony (i.e., the coordination or co-occurrence of affective states between individuals), across family triads consisting of mothers, fathers, adolescents. We differentiate positive affect (PA) synchrony from negative affect (NA) synchrony and examine their associations with adolescent well-being and distress. Distinguishing these two forms of synchrony matters because positive and negative emotions are thought to serve different interpersonal functions within relationships: fostering connection when shared positively, versus signaling a need for support or regulation when shared negatively [15,16]. To avoid confusion between these synchrony constructs and adolescents’ own affect, we use “PA synchrony” and “NA synchrony” specifically to refer to the degree of coordination in positive or negative affect across family members, as distinct from “PA” and “NA,” which refer to adolescents’ own individual-level positive and negative affect.

### Family systems theory

Adolescent development occurs within an intricate system of relationships [17]. Integral to this system are adolescents’ evolving relationships with core members of their family [18–21]. Family relationships are unique in their enduring nature, prominence in daily life, high emotional impact [6], and embeddedness within a larger dynamic system [19]. These relationships provide the most proximal impact on development and play a pivotal role in well-being throughout life [6,21]. Yet the specific relational needs that best support well-being appear to shift across developmental stages [22].

The family is more than a set of its individual members or even dyadic relationships. According to family systems theory, it is a complex system of interrelated components that interact in unique and dynamic ways [19]. Within the family, relationships are mutually influential and bidirectional in nature [23]. Through these relationships, individuals experience interdependence and interconnectedness [19]. Each family has a unique set of dynamics that are influenced by each individual member as well as the unique interrelated dynamics that compose the family system [19]. Of particular importance for adolescent development are adolescents’ evolving relationships with their parents, which are reshaped by concurrent biological and social changes [10] even as parents remain critically important to adolescent development [17].

Parents are a critical component of the family system. Parents influence the family through the individual relationships with their children as well as through the relationship they have with each other [24]. Prior research in this area has primarily focused on dyads in family research, particularly the mother-child dyad [25]. Although this research highlights the importance of the parent-child relationship on adolescent development [17], it neglects the role of fathers [26,27]. In the United States the majority of adolescents (68%) reside in a home with both their mother and father [7]. Fathers are playing an increasingly active role in the lives of their children [26]. Research has shown that high emotional closeness between fathers and adolescents has been linked to positive behavioral outcomes and reduced emotional distress in adolescents [26]. Conversely, father absence has been linked to poorer adolescent well-being and developmental outcomes, further underscoring the importance of the father-adolescent relationship [27].

Not only does a mother-child dyadic perspective neglect the role of fathers, but it also underestimates the structure of the family system. The family operates as a dynamic system of interrelated individuals and relationships, which can be represented as a network [19]. Not only are these relationships bidirectional in nature but they also influence and are influenced by the family context across time [28]. Family systems research has demonstrated the importance of understanding the family context in relation to children’s development [19,21,23]

A network perspective of the family provides a useful framework to analyze individual members of the system, the relationships between family members, as well as the dynamics of the system as a whole [19,25,29,30]. This comprehensive understanding of the family context provides the opportunity to analyze specific dynamics within families that have the potential to influence adolescent development and well-being [5,25].

### Family emotional dynamics

A critical aspect of the family system is the emotional dynamics within the system [4]. The emotional dynamics within a family system include how emotions are experienced individually, within relationships, and across the system as a whole [3]. These dynamics are influenced by the quality of family relationships, such as the attachment quality in parent-child relationships and the intra-parental relationship, as well as the emotions expressed within the family [3,4]. Family emotional expressivity includes not only the range of emotions, from positive and negative, but also the prevalence and intensity of emotions expressed by all members of the family [4].

The emotional dynamics of a family system demonstrate the interconnectedness of emotional processes of the members within the family [16,31]. For example, the emotions of one family member can influence the emotions of others as well as the system as a whole. This interdependence is especially important during adolescence, when youth are navigating heightened emotional reactivity, alongside growing autonomy, increasing self-regulation, and changing relationships with parents [10,11,14]. In this context, family emotions are not simply individual experiences occurring in parallel; rather, emotional experiences are embedded in ongoing patterns of interaction among adolescents and their parents [12,16,25].

Consistent with this perspective, family emotional dynamics have been linked to adolescents’ development and well-being [2–4]. To illustrate, research has shown that the emotional climate of the family impacts the development of emotion regulation in adolescents [3,4]. Other studies have examined the link between adolescent depression and the emotional dynamics within families, finding that families of depressed adolescents show distinct affective patterns compared to families of non-depressed adolescents [2,12]. For instance, Bodner et al. [2] found that families of depressed adolescents expressed more anger over time than families of non-depressed adolescents. Positive family dynamics have similarly been linked to adolescent well-being: for example, day-to-day parent-adolescent connectedness predicts greater daily well-being and eudaimonia for both parents and adolescents [5], and warm, supportive parent-adolescent relationships are associated with better adolescent emotion regulation and adjustment [32]. Together, this work suggests that family emotional dynamics are meaningfully related to adolescent adjustment and may provide an important window into family-level processes associated with well-being and distress.

Family emotion dynamics are well-suited to be examined from a dynamic systems perspective due to the interconnectedness of these emotions as they unfold across time. From this view, individual members’ affective states are not isolated events, but rather represent micro-processes of the broader family emotional system. Momentary affective states may also dynamically shape broader family dynamics because emotions play an integral role in interpersonal relationships and family interactions [25]. Thus, understanding family emotional dynamics requires attention not only to what individual family members feel, but also to how emotions co-vary, cluster, and become coordinated across the family system.

EMA studies have increasingly captured family emotions as they occur in daily life, providing a foundation for examining how adolescents’, mothers’, and fathers’ emotional states fluctuate across time [25,33,34]. Dynamic network analysis offers such a framework by representing emotions as components of a broader system rather than as isolated individual states. In this approach, emotions are modeled as nodes and their associations over time as edges, making it possible to examine how affective states cluster, interrelate, and organize within and across people [9,30]. In the context of family research, this approach allows the family to be conceptualized as a dynamic emotional network in which adolescents’, mothers’, and fathers’ affective states are linked in ongoing and reciprocal ways. As such, dynamic network analysis is well suited to study emotional coordination and synchrony in family life as they unfold across time [9,25].

### Family emotional synchrony

Emotions are inherently interpersonal, emerging within relationships and shaped by social context, covarying with the affective states of others over time. A growing body of work has examined *emotional synchrony*, broadly defined as the coordination or co-occurrence of affective states between individuals, as a key property of emotional systems [12,16,25,31,35]. Importantly, emotional synchrony acknowledges the interpersonal nature of emotions that occur within the bi-directional context of relationships. In this paper, we conceptualize emotional synchrony as the degree to which family members’ emotions are in sync, or how their emotions change together over time. Specifically, we quantify synchrony by measuring the consistency and strength of emotional connections among family members (adolescent, mother, father) over time across daily life.

Synchrony may also differ according to emotional valence. Positive emotions may spread relatively easily among family members through shared enjoyment and reinforcement, reflecting the interconnected nature of family emotional systems [16], whereas negative emotions may be more selectively regulated, buffered, or contained within specific relationships to protect other family members from distress [3,4]. These valence-specific dynamics suggest that synchrony in positive affect and synchrony in negative affect may reflect distinct interpersonal processes rather than a single, unified construct. Accordingly, throughout this paper we examine PA synchrony and NA synchrony as separate, though related, family-level processes.

Emotional synchrony within families is closely linked to the broader process of co-regulation, in which one family member helps modulate another’s emotional state through responsive, attuned interaction [35]. From an attachment perspective, co-regulation between parents and children is considered one of the primary functions of the parent-child bond, allowing children to draw on a caregiver’s regulatory capacity before they are able to regulate independently [20]. Although co-regulation has historically been studied in early childhood, parents continue to scaffold their children’s emotion regulation well into adolescence, even as adolescents gain autonomy and increasingly turn to peers [11]. Synchrony offers one behavioral signature of this coregulatory process: when family members’ affective states rise and fall together, this may reflect not only shared emotional experience but also the ongoing regulatory influence that parents and adolescents exert on one another [16,35].

Research on emotional synchrony has primarily focused on dyadic relationships, including romantic partners and parent-child dyads [31]. More recently however, research on emotional synchrony has begun to examine triadic emotion dynamics among mothers, fathers, and adolescents [2,12,25]. Hollenstein and colleagues [12] analyzed the affective patterns of mother-father-adolescent triads during three structured interactions and found that families of depressed adolescents displayed a broader range of affect, experienced less predictable affective patterns, and spent less time in synchronous affective states Similarly, Bodner et al. [2] found that affective daily interactions were associated with adolescent depression, further suggesting that triadic-level emotion dynamics are meaningfully related to adolescent well-being and mental health. Veenman and colleagues [25] analyzed four affect states—happy, sad, irritated, and relaxed—and demonstrated how adolescents,’ mothers’, and fathers’ affect states were related both concurrently in the moment and over time. Together, these studies demonstrate the added value of using a triadic perspective for family systems research. Family emotional dynamics depend not only on how one parent and child are emotionally linked, but also on how multiple family members are coordinated within the same emotional system. Triadic approaches therefore capture family-level patterns of emotional organization within the mother-father-adolescent system that may be missed when families are only examined as three separate members or as combinations of isolated dyads.

### The current study

Adolescence is a period of significant emotional change that carries heightened risk for mental health difficulties [10,14], but it is equally a period of opportunity, marked by growing emotion regulation skills, deepening relationships, and a developing foundation for long-term well-being [10]. The family is integral to both the risks and opportunities of adolescent development [6], with family emotional dynamics linked to a range of adolescent outcomes [2–4,12,25]. Thus, the family’s emotional environment presents a potential area for enhancing the physiological and psychological well-being of young individuals [36]. Although prior research has established the importance of all members of the family for adolescent development and well-being, few studies have adopted a network perspective and incorporated triads into the research [12,16].

Prior work on family emotions has often relied on single-time-point assessments, laboratory interactions, or structured scenarios [12]. These approaches are limited in their ability to capture how families’ emotions unfold in daily life. We adopt an ecological momentary assessments (EMAs; [8]) approach, which provides more ecologically valid data by repeatedly sampling participants’ current experiences in real time and in their natural environments. The EMA approach reduces reliance on retrospective recall and allows researchers to examine emotional microprocesses as they vary across time and context [8,37,38]. Thus, the EMA approach is especially well-suited for capturing emotional synchrony as it occurs in the dynamic, interpersonal, and embedded contexts of everyday family life.

However, EMA alone does not model the interdependence of emotions across family members. To address this, the current study applies dynamic network modeling [9,30] to examine the real-time emotional interactions within nuclear family networks. Specifically, we will examine the temporal patterns of emotional synchrony—categorized as positive and negative affect synchrony—among family members. We also test whether family emotional synchrony is associated with adolescent daily well-being and mental health outcomes. Well-being is operationalized using the PERMA framework, comprising five elements: positive emotion, engagement, relationships, meaning, and accomplishment [39], which we examine individually rather than as a composite score. Mental health outcomes are operationalized as perceived stress, depression, and anxiety. Throughout, we use family emotional synchrony as the overarching construct, distinguishing PA synchrony and NA synchrony, each assessed at the overall (between-family) and momentary (within-family) level.

The present study will address the following research questions.

RQ 1: What are the temporal patterns of emotional synchrony among family members?

a. Do families show similar patterns of emotional synchrony across positive emotions and negative emotions?
b. To what extent do family triads vary in the structure and strength of emotional synchrony, and what characterizes this heterogeneity across families?

RQ2: Is family emotional synchrony associated with improved adolescent well-being and reduced stress, depression, and anxiety?

a. Is overall family emotional synchrony associated with improved overall psychological well-being in adolescents?
b. In moments when families experience higher levels of emotional synchrony, do adolescents report higher well-being outcomes?
c. Do these effects hold after controlling for adolescent average levels of PA & NA?

Based on prior research on emotional synchrony and family emotion dynamics, we hypothesize the following. First, we hypothesize that temporal patterns of emotional synchrony will exist within families throughout their daily lives, aligning with current research on affective patterns within family triads demonstrating emotional synchrony as an important component of the dynamic family emotional system [12,25]. Next, we further hypothesize that family emotional synchrony will be associated with improved adolescent well-being outcomes of daily well-being and self-reported ‘stress’, consistent with several studies that have linked family emotional dynamics with adolescent outcomes [2–4,25] including Hollenstein et al. [12] that found an association between reduced family emotional synchrony with adolescent depression.

## Method

The study (“Study of Network Dynamics of Family Emotions”) was approved as exempt by the Institutional Review Board at Claremont Graduate University, IRB protocol #4673. All research was performed in accordance with the Declaration of Helsinki.

### Openness and transparency

This study was not preregistered. Study materials, data, and analysis code are available on OSF (https://osf.io/j5duq/). All analyses were conducted using R (version 4.4.4). DynEGA was applied using the *EGAnet* package (version 2.4.0; Golin(version 2.4.0; [40])ts were visualized using the *ggpubr* (version 0.6.0), *GGally* (version 2.2.1), and *ggplot2* (version 3.5.1) packages in R. Multilevel modeling was applied using *lme4* (version 3.1–163; Bat(version 3.1–163; [41])

### Participants

See Table 1 for demographic details for age and race/ethnicity for this sample of *N* = 40 families (i.e., *N* = 120 individuals). Child gender breakdown: male *n* = 18 (45%), female *n* = 19 (47.50%), non-binary *n* = 1 (2.5%). Most of the families came from relatively high socioeconomic backgrounds. The combined annual income for the families was as follows: $30,000–$59,999 = two families (5%), $90,000–$119,999 = five families (12.5%), $120,000+ = 32 families (80%), and one family preferred not to respond. Most families had parents with high levels of education: the majority had attained a bachelor’s degree or higher (mother = 80%, father = 72.5%). We aimed to recruit heterosexual couples to reduce heterogeneity in family structure; sexual orientation itself was not directly assessed beyond this recruitment focus.

**Table 1:** Age and race/ethnicity sample characteristics of *N* = 40 family triads.

Characteristic		Child	Dad	Mom
Age (years)		*M* = 14.55 (*SD* = 1.32) Range: 12–17	*M* = 49.28 (*SD* = 5.06) Range: 40–59	*M* = 46.73 (*SD* = 4.62) Range: 37–58
Race/Ethnicity				
	American Indian or Alaska Native	—	—	1 (2.5%)
	Asian or Asian American	5 (13.2%)	5 (12.5%)	9 (22.5%)
	Black or African American	1 (2.6%)	4 (10%)	2 (5%)
	Hispanic, Latino, or Spanish Origin	3 (7.9%)	8 (20%)	6 (15%)
	Middle Eastern or North African	1 (2.5%)	1 (2.5%)	1 (2.5%)
	White or European American	14 (36.8%)	18 (45%)	17 (42.5%)
	Mixed Race / Multiracial	14 (36.8%)	2 (5%)	4 (10%)
	Prefer not to answer	—	2 (5%)	—

### Procedures

Participants were recruited through convenience and snowball sampling using social media and word of mouth. Recruitment for the study occurred from October 2024 until April 2025 (until a full sample of 40 families was achieved). Inclusion criteria were that (1) all three family members resided at the same U.S. address, (2) English proficiency from all members to complete surveys, and (3) smartphone ownership and ability to download apps and answer surveys (i.e., each member, including the adolescent, had their own personal smartphone). Interested parents completed a screener survey, were contacted by the researchers, and were then scheduled for an online onboarding meeting with the research assistant and all three family members present. Eligible participants were then provided online informed consent forms (parents) and informed assent forms (adolescents).

After obtaining informed consent (from parents) and assent (from the adolescent), family participants began the research study (see Fig 1). In Phase 1, participants completed an Introductory Assessment via Qualtrics, a battery of questionnaires that included sociodemographic questions and trait-level measures (not analyzed in the current study). They also configured their smartphones with Avicenna (EMA software) for the EMA data collection phase. During this phase, family participants specified their time preferences for receiving EMAs from a preset list, ensuring that all family members received EMA signals within the same time window. These preset windows were intentionally scheduled outside a typical adolescent’s school day to prevent EMA prompts from disrupting academic activities. Specifically, the morning time windows were set to “before school” periods (6–7:30 a.m.; 6–8 a.m.; 7–9 a.m.), the afternoon windows were set for typical “after school and extracurricular activities” periods (3–4 p.m.; 3–5 p.m.; 4–6 p.m.), and the evening window was set for “before bedtime” periods (6–9 p.m.; 7–10 p.m.). In Phase 2, participants completed three EMAs at three semi-random times (morning, afternoon, and evening) over seven consecutive days in the main study. In Phase 3, all participants completed an Exit Assessment (additional trait-level measures, not used in the current study) the day after completing the EMA phase of the study. Family participants were compensated up to $150 per person (i.e., $450 per family of three), proportionate to the number of assessments they completed.

**Fig 1:**
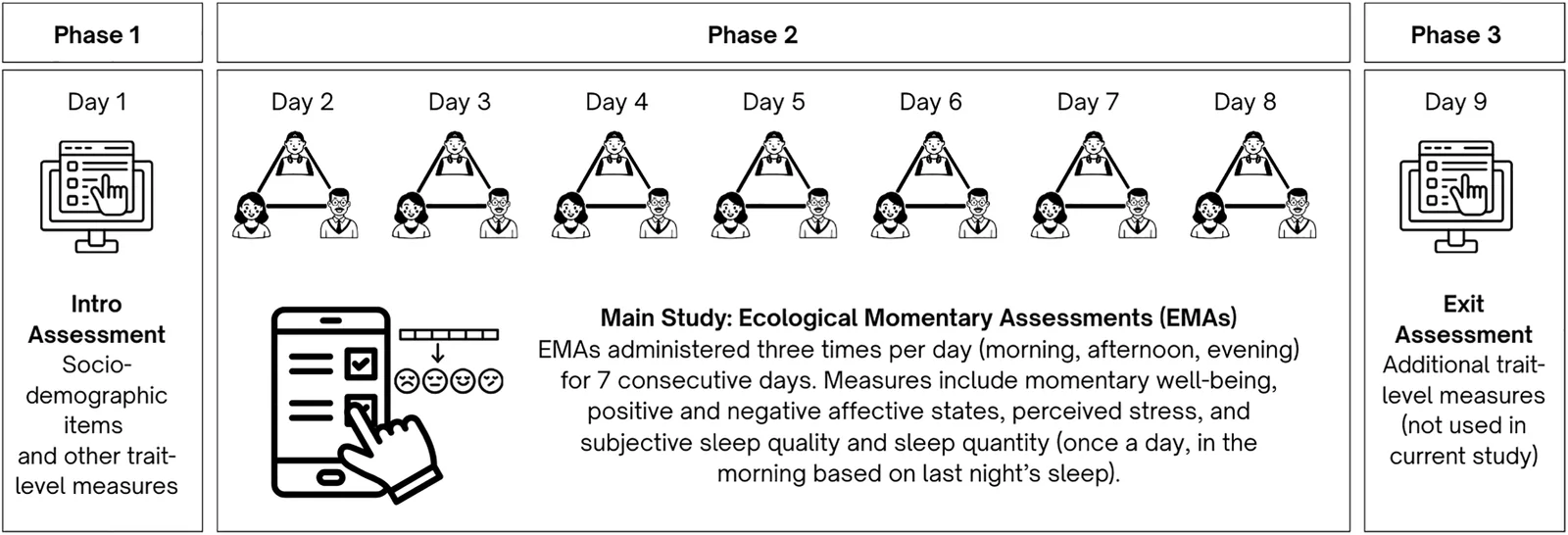
Overview of study schedule. The figure depicts the participant study schedule from Day 1 to Day 9, after all three members have consented to the study, participated in an online onboarding session with a research assistant.

### Measures and data

For more details on descriptive statistics (e.g., means, SDs) for the measures used, see Supporting information.

#### Momentary affect.

Momentary affect was assessed via self-report at each time point (three times per day for one week) using the Subcomponents of Affect Scale (SAS; [42]), which includes an 18-item affect scale with nine positive affect (PA) and nine negative affect (NA) items. The PA items were: Lively, Full-of-Pep, Energetic, Happy, Pleased, Cheerful, At Ease, Calm, and Relaxed. The NA items were: Sad, Depressed, Unhappy, On edge, Nervous, Tense, Hostile, Resentful, and Angry. Participants were prompted, “Please indicate the extent to which you feel the emotion listed below right now,” and had response options ranging from 1 (*Very slightly/Not at all*) to 5 (*Extremely*). The current study focuses on the momentary affect data from all three family members when constructing dynamic emotion networks. The SAS was selected because it is a validated state affect measure with demonstrated stability across repeated daily assessments, well suited to the present EMA design, and because it captures a wider range of affective states than many other affect scales. Internal consistency of the SAS subscales was evaluated using multilevel coefficient omega (ω), with repeated EMA assessments nested within participants and all family members pooled [43]. Within-person and between-person reliability estimates (respectively) were ω = .86 and ω = .97 for Positive Affect items, and ω = .84 and ω = .95 for Negative Affect items, showing good reliability at the within- and between-levels.

#### Adolescent momentary well-being and stress.

Adolescents’ momentary well-being was operationalized using Momentary PERMA (mPERMA; [44]), an adapted version of the PERMA-Profiler [45] validated for intensive longitudinal designs and for measuring elements of well-being in daily life. *m*PERMA measures the various building blocks of participants’ multifaceted well-being [39,46]. For each item, participants were prompted with “Right now…” and a slider scale ranging from 0 (*not at all*) to 100 (*completely*). There were three items based on each element of PERMA for a total of 15 items: Positive emotions (e.g., “I am feeling joyful”), Engagement (e.g., “I was absorbed in what I was doing”), Relationships (e.g., “I feel helped and supported by others”), Meaning (e.g., “I have a sense of direction in my life”), and Accomplishment (e.g., “I am making progress towards accomplishing my goals”). The five mPERMA elements were examined individually as separate momentary outcomes rather than combined into a single composite well-being score. Each item was presented in a randomized order on participants’ smartphones to reduce bias from order effects. Although the mPERMA Positive emotion items and the SAS positive affect items both capture positive affective experience, the two scales were designed for distinct purposes, the SAS as a general momentary affect measure and mPERMA as a multidimensional profile of momentary well-being, and were treated as conceptually and analytically distinct constructs in the present study. For the reliability of adolescent participants’ scores on mPERMA, reliability was assessed at the within- and between-person levels [43]. Within-person and between-person omega (ω) estimates (respectively), were .84 and .88 for Positive emotion, .73 and .88 for Engagement, .69 and .92 for Relationships, .74 and .98 for Meaning, and .82 and .98 for Accomplishment, showing good reliability for both levels.

Adolescent stress was assessed via self-report at each time point. They were prompted, “How stressed do you feel right now?” and responded on a slider scale ranging from 0 (*Not at all*) to 100 (*Extremely*).

#### Trait-level well-being and mental health measures.

At the trait-level, adolescents’ overall well-being was assessed using the PERMA-Profiler [45]. Adolescents’ overall mental health was assessed using the Perceived Stress Scale (PSS; [47]) and also the Depression, Anxiety, and Stress Scale (DASS-21; [48]). Lastly, adolescents’ general positive and negative affect was assessed using the 20-item Positive and Negative Affect Schedule (PANAS; [49]). The Cronbach’s αs for Trait-Level outcomes are as follows: P = .87, E = .49, R = .63, M = .76, A = .60, perceived stress = .76, depression = .89, anxiety = .62, DASS-21 stress = .83., overall (PANAS) positive affect (PA) = .85, and negative (NA) = .89.

#### Data.

The initial dataset comprised *N* = 2,382 assessments, which were cleaned to address missing data (i.e., entire rows), duplicate assessments, and inconsistent timestamps (app glitches). The cleaning process removed a negligible amount of EMAs (*n* = 17; < 1%). The final dataset comprised *N* = 2,365 EMAs, each containing momentary affect ratings from all three family members over one week of study. The overall compliance rate was 94%, based on a possible total (100% compliance) of 2,520 EMAs (40 families × 3 family members × 3 timepoints × 7 days). Breaking this down further, the total EMAs for each family member were adolescent *n* = 755 (90% compliant; out of 840 total possible), father *n* = 796 (95% compliant), and mother *n* = 814 (97% compliant). Across individuals, the mean number of completed EMAs was *M* = 19.71 (SD = 2.22, range = 9–21). Other data on sociodemographic characteristics (e.g., age, gender) were obtained from all participants’ first introductory assessment via Qualtrics.

### Data analysis plan

#### Creating family emotion networks for positive and negative affect.

Dynamic networks were created for each family triad’s positive and negative emotional systems. The structure of these networks can then be analyzed to assess levels of synchrony overall (between-family levels) and in the moment. For a conceptual illustration of how the network structures were constructed and hypothetical examples of a high synchrony and a low synchrony family, see Fig 2. Each node (circle) represents a family member and their aggregated state affect scores (e.g., positive affect), and each edge (line) connecting the nodes represents the degree of association (i.e., synchrony) between them. Green edges represent positive associations and red edges represent negative associations. The panels labeled “Aggregate” depict the total “stacked” partial correlations over time. Family A demonstrates high synchrony in their state affect scores over time, as evidenced by the consistent green edges across the days of the study between all three members. Family B demonstrates a low synchrony score due to negative red edges and, at times, the absence of edges (zero association) between members across the study days.

**Fig 2:**
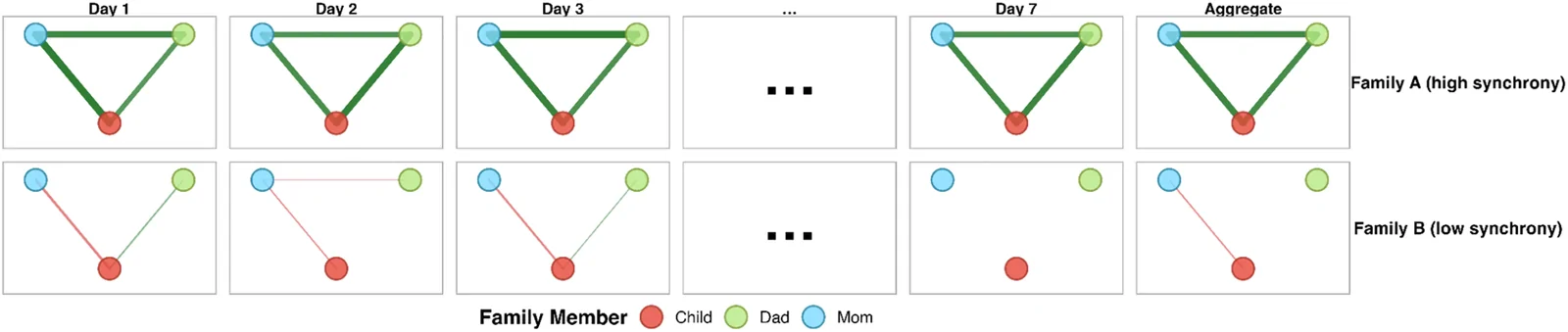
Conceptual Illustration of Network Structures for High and Low Synchrony Family Triads. The figure depicts a conceptual illustration of affect networks involving two families (Family A, top row; Family B, bottom row) over seven days of the study. *Conceptual Illustration of Network Structures for High and Low Synchrony Family Triads.*

#### Constructing family emotion networks using dynamic exploratory graph analysis.

The networks and dimensions for the affect data at the sample-level and individual-family triad levels were estimated using dynamic exploratory graph analysis (dynEGA; [9]), an extension of exploratory graph analysis [50,51]. First, to estimate networks for the sample and for individual family triads within the sample, the first-order derivatives of all affect variables (positive and negative) within each family were computed using the Generalized Local Linear Approximation (GLLA; [52]). This resulted in an *n*-by-54 matrix for each family, where the rows correspond to the *n* first-order derivatives and the columns correspond to each family member’s affect variables. Next, the individual-family triad networks were estimated for each family’s first-order derivative *n*-by-54 matrix. The sample-level network was estimated by “stacking” the derivative matrices such that the rows represented each family’s first-order derivatives consecutively and the columns represented every SAS item from the full scale (rather than using composite scores or subscales). For each family case, the graphical least absolute shrinkage and selection operator (GLASSO; [53]) with the extended Bayesian information criterion (EBIC; [54]) was used for model selection to estimate each network. Missing data were handled using Kalman smoothing to generate estimates for the missing observations while preserving the original time scales [55,56]. The lower-order Louvain algorithm [57] was applied for each network. The final network for each family represents the SAS items as nodes and the partial correlations of the first-order derivatives as edges. For more details, see the Supporting Information for technical details on the dynamic network and dimension estimation (*S1.2 Network and Dimension Estimation)*, the Louvain algorithm (*S1.3 Louvain Algorithm*), methods for handling missing data (*S1.4 Missing Data and Noise Jittering*), and zero-variance derivatives (*S1.5 Zero-Variance Derivatives*).

In each dynamic network (for PA and NA), the nodes (circles) represent components (in this case, SAS items at the lower level, aggregated to the family member at the higher level) and edges represent how two components change together across time, given all other components. Thus, conceptually, *positive emotional synchrony* would indicate that two nodes have similar scores that rise and fall together across time, whereas a *negative emotional synchrony* would indicate an inverse relationship between two nodes (in which as scores for one node rises, the other falls) together across time. To illustrate conceptually how family emotion networks were constructed in dynEGA in a hierarchical fashion, see Fig 3 for an empirical example for one family triad. For this empirical example, the child and mother have strong, positive synchrony in positive affect (thick green line) where their PA rise and fall together across time; the child and father have weak positive synchrony in positive affect (thin green line); and the mother and father have a negative (i.e., inverse) synchrony in positive affect (red line) where PA rises for one when it falls for the other. For negative affect, mother and child show weak positive synchrony (thin green line), whereas all other relational pairings show negative synchrony (red lines).

**Fig 3:**
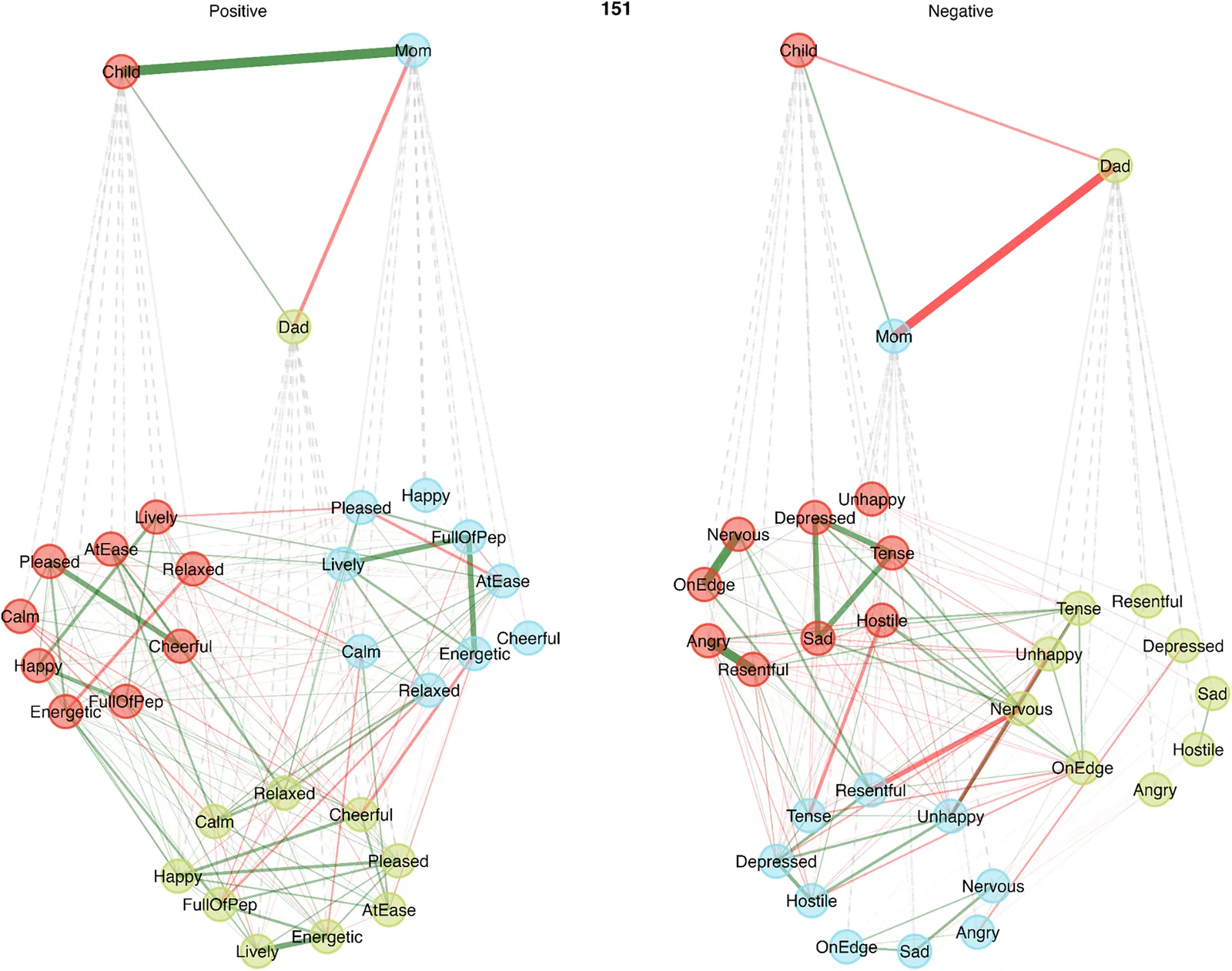
Hierarchical representation of affect synchrony for one family triad. An empirical example of a family triad’s positive (left) and negative (right) affect variables over time is shown as an interconnected, two-level dynamic network. In the bottom level, nodes (circles) represent the affect variables for each family member, node colors correspond to family members, and the edges (lines) represent partial correlations between nodes. Green edges represent positive regularized partial correlations with the thickness indicating magnitude, while red edges represent negative associations (as one node increases over time, the other paired node decreases). In the top level, a visual representation is shown that takes the synchrony aggregates of each pairwise relationship (child–mom, child–dad, mom–dad) and maps it where each node is the family member and each edge is the aggregated emotional synchrony scores (ranging from negative to positive; thickness indicating magnitude).

#### Assessing family emotional synchrony.

Definitions of synchrony vary. For technical details on emotional synchrony metrics, see *S1.6* (*Assessing Synchrony of Family Emotions*) in Supporting Information.

***Level 2 (between-level) family emotional synchrony.*** From a dynamic network framework perspective, we conceptualize overall family emotional synchrony in the current study as the overlap in the rates at which two (or more) variables change together over time [58]. To quantify overlap for each network, we computed the absolute value of the weighted topological overlap (wTO; [59]) for each pairwise node combination in the network.

wTO values represent the extent to which two variables change together and similarly change with other variables. For each pair of nodes, the wTO represents general synchrony among the variables without directionality (i.e., overall magnitude of synchrony), where smaller values closer to 0.00 indicate less synchrony and greater values indicate greater synchrony. Thus, in this study, family emotional synchrony is operationally defined as the total wTO values for affect state nodes over time within family networks. It is computed between each pair of variables with one node for each family member’s emotional state (e.g., child-happy with mother-happy, child-happy with dad-happy, child-happy with mother-calm). When taken together, the sum measures the extent to which the family, as an emotional system, is synchronized. Higher positive (green) synchrony values indicate that the family’s system of affective states change in similar ways over time (e.g., PA child and PA mom in Fig 3); negative (red) indicate that the change occurs in opposing ways over time (e.g., PA up for mom and PA down for dad in Fig 3). We specifically operationalized *positive emotional synchrony*, which considers nine positive affect variables (happy, pleased, etc.), as the sum of the pairwise wTO values for each family triad. We calculated *negative emotional synchrony* in the same manner for the negative affect variables.

***Level 1 (momentary-level) family emotional synchrony.*** Momentary family emotional synchrony was operationalized as the degree of similarity in concurrent affect ratings among family members at each EMA prompt. For each of 18 SAS items (9 PA; 9 NA), absolute differences were computed for each dyad within the triad (child–mother, child–father, father–mother). These item-level discrepancy scores were averaged within valence to yield dyad-level discrepancy indices, which were then averaged across the three dyads to obtain family-level momentary discrepancy scores. To facilitate interpretation such that higher values reflected greater synchrony, discrepancy scores were reverse-coded using the formula. Thus, this resulted in momentary PA synchrony and momentary NA synchrony variables ranging from 0 to 4, with higher values indicating greater similarity in family members’ affect at that prompt. When data were partially missing, scores were computed using available item and dyad information (i.e., pairwise deletion within row means).

#### Predicting adolescent outcomes using emotional synchrony metrics.

To examine whether family emotional synchrony predicts adolescent well-being and stress, we estimated a series of single-level regression models (RQ2a) and two-level linear mixed-effects models (RQ2b). Detailed model specifications, diagnostics (e.g., multicollinearity, intraclass correlations), and full equations are reported in *S1.7 Multilevel Modeling* in Supporting Information.

For RQ2a, we estimated multiple linear regression models in which adolescents’ trait-level outcomes (the Five PERMA elements (Positive emotions, Engagement, Relationships, Meaning, Accomplishment) examined individually, along with, perceived stress, and DASS-21 facets) were regressed on overall family positive and negative affect synchrony. Overall synchrony was operationalized as the non-signed sum of wTO values aggregated (for PA and NA separately) across the study period and grand-mean centered.

For RQ2b, we estimated two-level linear mixed-effects models with momentary observations nested within individuals. Momentary adolescent outcomes (positive emotions, engagement, relationships, meaning, accomplishment, and stress) were modeled as a function of both within-person and between-person predictors. At Level 1, momentary positive and negative affect synchrony (person-mean centered) were included as time-varying predictors, capturing within-person fluctuations in synchrony across moments. At Level 2, overall family positive and negative affect synchrony (grand-mean centered wTO indices) were included as between-person predictors.

For RQ2c, we re-estimated the RQ2a and RQ2b models with adolescents’ general affect levels added as covariates, to test whether family emotional synchrony predicted adolescent outcomes beyond adolescents’ own typical or concurrent affect. In the RQ2a models, adolescents’ overall trait positive affect and trait negative affect were added as additional person-level predictors. In the RQ2b multilevel models, adolescents’ momentary positive affect and negative affect (person-mean centered) were added as additional Level 1 covariates alongside momentary family synchrony.

All multilevel models included random intercepts for adolescents to account for the non-independence of repeated observations. Random slopes were not specified, as we did not hypothesize systematic between-person variability in the within-person associations over the short (1-week) sampling period. Models were estimated separately for each outcome variable.

## Results

### Temporal patterns of emotional synchrony among family members

Generally, across all families, there was an overall greater synchrony in positive affect items (*M* = 8.09; SD = 3.87) than in negative affect items (*M* = 6.22; *SD* = 3.40). In the moment, there was greater synchrony in negative affect (*M* = 3.52, *SD* = 0.46) than in positive affect amongst the triad members. See Table 2 for descriptive statistical details.

**Table 2:** Patterns of family emotional synchrony based on weighted topological overlap values.

Network Metric	*M* (*SD*)	Range	Median	Skew	Kurtosis
Overall PA Synchrony	8.54 (3.48)	0–12.56	9.57	–1.33	0.65
Overall NA Synchrony	6.69 (3.06)	0–10.72	7.52	–0.82	–0.52
Momentary PA Synchrony	2.86 (0.43)	0.89–4.00	2.89	–0.53	0.62
Momentary NA Synchrony	3.52 (0.46)	0.22–4.00	3.63	–1.57	4.07

Overall PA and NA Synchrony scores (Level 2) are operationalized as absolute value of the weighted topological overlap values for each pairwise node combination in the respective valenced network (*N* = 40). Momentary PA and NA Synchrony scores (Level 1) are operationalized as the absolute differences were computed for each dyad within the triad for every moment (*N* = 2,326).

Specifically for positive affect (PA) networks, the majority of families had average synchrony values within ±1 SD of the mean (*n* = 31; 77.50%), with two families having high overall PA synchrony (5%) and seven families having overall low PA synchrony (17.50%). A similar pattern emerged for the negative affect (NA) networks. For NA, the majority of families had average synchrony values that were within ± 1 SD of the mean (*n* = 28; 70%), with five families having high NA synchrony (12.50%) and seven families having low NA synchrony (17.50%). See Fig 4 for a visual depiction of the distribution of positive and negative affect synchrony scores.

**Fig 4:**
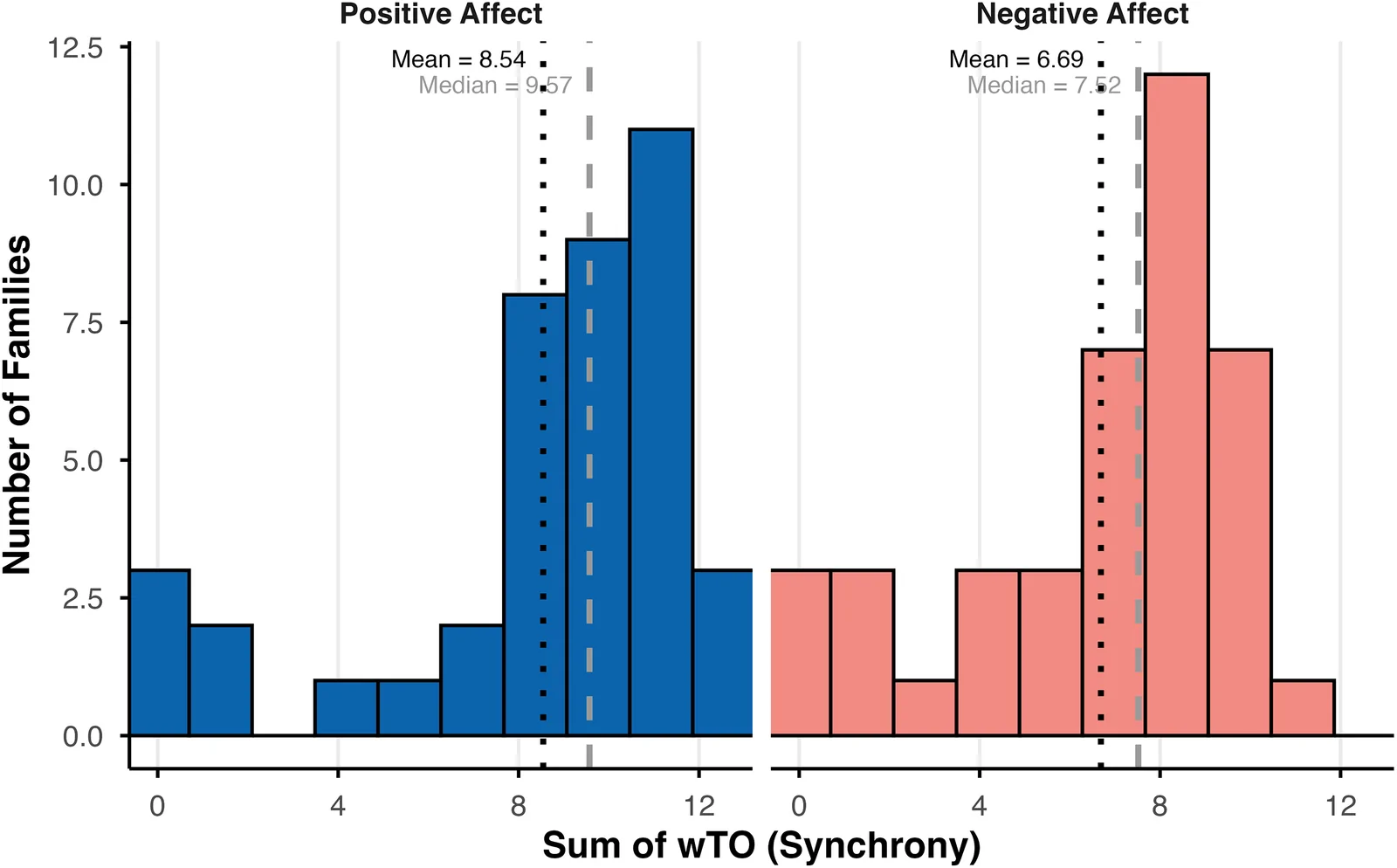
Distribution of family-level emotional synchrony values for positive and negative affect. The blue histogram on the left represents family-level synchrony values for positive affect, while the pink histogram on the right is for negative affect.

There was considerable variability in the synchrony patterns of positive and negative affect for each family triad. See Fig 5 for the aggregated positive and negative affect networks for all forty families (which can be fully depicted as in Fig 3). Within the same family, there are varying patterns over time in emotional synchrony for each paired-family member and for the positive and negative affect items. For example, FID #151 Positive Network—throughout the week, mom reports increased positive affect, and the child does as well. Yet for that same network and family triad, as mom reports increased positive affect, dad reports decreased positive affect. An example of a family with high overall emotional synchrony is FID #166 across both positive and negative affect items, as evidenced by thick green edges amongst all family members. Some families (e.g., FID #s 172, 186) have zero or near-zero synchrony (very thin or nonexistent edges) in positive and negative emotions among family members, indicating low synchrony in their emotional experiences as a family triad throughout the week.

**Fig 5:**
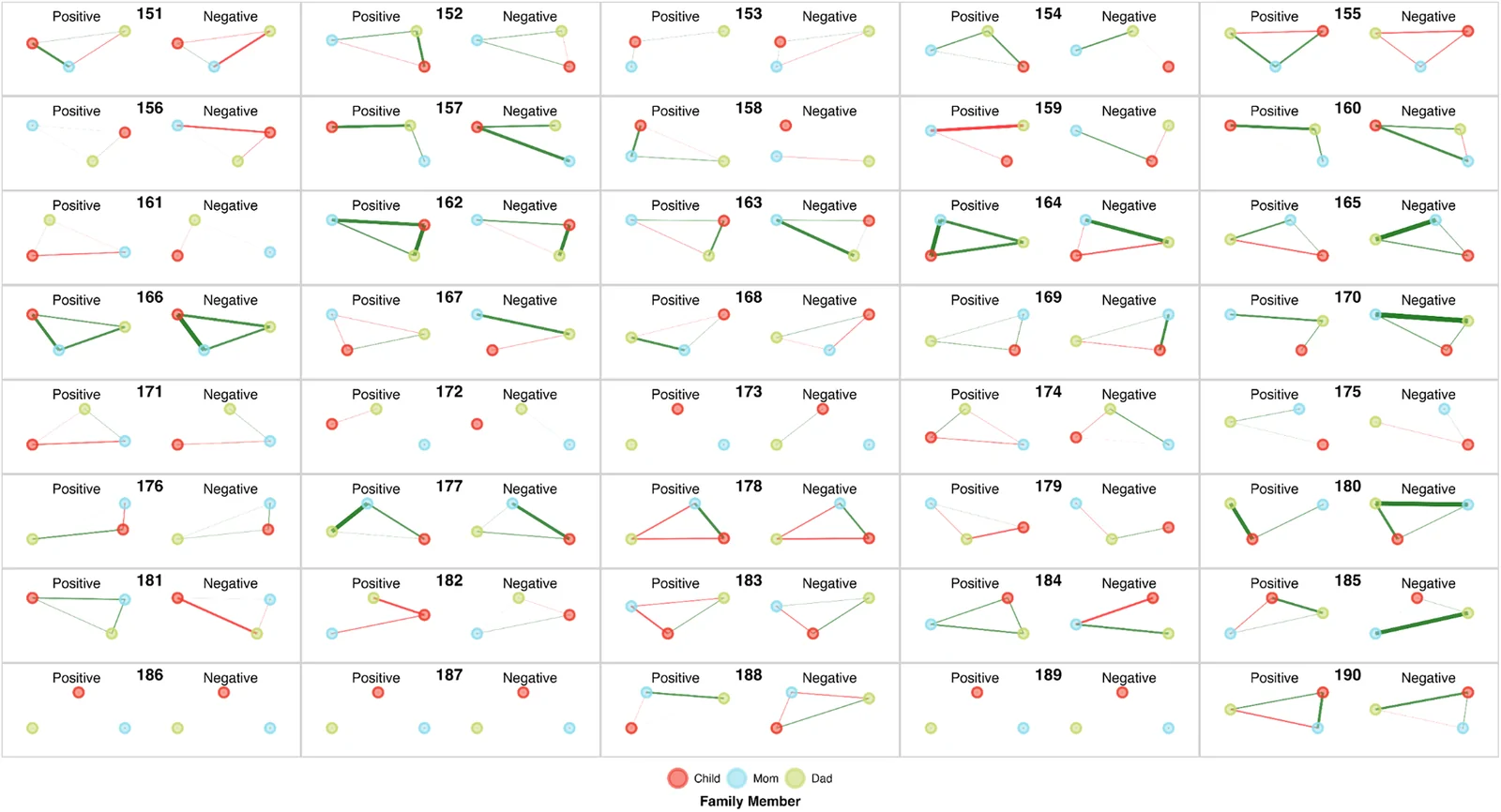
Aggregated positive and negative affect networks for forty families. The figure depicts the aggregated positive and negative affect synchrony for all 40 families. Nodes (circles) represent each family member. Edges (lines) represent the absolute sum of weighted topological overlap (wTO) values between each of the affect variables for each family pair (child-mom, child-dad, and dad-mom). wTO values were aggregated separately for positive emotions and negative emotions. The thicker the edge, the larger the absolute value for the total sum of all affect variables between that family pairing. For each Family ID number (#s 151–190), plotted below is the aggregated positive affect network on the left, and the aggregated negative affect network on the right. Green edges indicate greater synchrony in affective variables for that pairing; for example, FID #151 Positive—mom reports increased positive affect, and the child does as well. Red edges indicate less synchrony; for example, FID #151 Positive—as mom reports increased positive affect, dad reports decreased positive affect. An example of a family with high overall emotional synchrony is FID #166 across both positive and negative affect items.

The distribution of family triad structures differed modestly between positive affect (PA) and negative affect (NA) networks. Triad classifications were computed separately for PA and NA networks using the non-signed, summed wTO values among child, mother, and father. Dyadic connections were considered present when |wTO| > .25. *Closed* triads indicate that *all three* dyadic connections (child–mother, child–father, and mother–father) were present. *Open* triads indicate that exactly *two of the three* possible dyadic connections were present. *Disconnected* triads indicate that *one or zero* dyadic connections were present. For PA networks, disconnected triads were the most prevalent (37.50%, *n* = 15), followed by open triads (35.00%, *n* = 14) and closed triads (27.50%, *n* = 11)—see Table 3 for details. In contrast, NA networks showed an even greater predominance of disconnected triads (45.00%, *n* = 18), with open triads comprising 35.00% (*n* = 14) and closed triads representing the smallest proportion (20.00%, *n* = 8). Comparatively, fully connected (closed) structures were more common in PA than in NA networks, whereas disconnected configurations were more frequent in NA networks. Overall, these patterns indicate that family emotional networks tend to be less cohesively interconnected in the context of negative affect relative to positive affect.

**Table 3:** Proportion of family triad structures in positive and negative affect networks.

Affect Valence	Triad Structure	*N* (%)	FamilyIDs
Positive	Closed	11 (27.50%)	155, 162, 163, 164, 165, 166, 178, 181, 183, 184, 190
	Open	14 (35%)	151, 152, 154, 157, 158, 160, 169, 170, 174, 176, 177, 180, 182, 185
	Disconnected	15 (37.50%)	153, 156, 159, 161, 167, 168, 171, 172, 173, 175, 179, 186, 187, 188, 189
Negative	Closed	8 (20%)	155, 160, 165, 166, 170, 178, 180, 188
	Open	14 (35%)	152, 156, 157, 162, 163, 164, 165, 168, 169, 174, 177, 183, 184, 190
	Disconnected	18 (45%)	151, 153, 154, 158, 159, 161, 171, 172, 173, 175, 176, 179, 181, 182, 185, 186, 187, 189

Dyadic connections were considered present when |wTO| > .25. *Closed* triads = all three dyadic connections were present; *open* triads = two dyadic connections; *disconnected* = one or zero dyadic connections.

### Associations with overall adolescent health and well-being outcomes

Family-level affect synchrony demonstrated differential associations with adolescent well-being at the person level. See Table 4 for details. Greater family PA synchrony was significantly associated with higher adolescent Positive emotions (*b* = 0.37, *SE* = 0.16, 95% CI [0.04, 0.70], *p* = .027), Engagement (*b* = 0.34, *SE* = 0.12, 95% CI [0.10, 0.58], *p* = .006), and Relationships (*b* = 0.33, *SE* = 0.14, 95% CI [0.04, 0.62] *p* = .028), with nonsignificant associations for Meaning and Accomplishment (*p*s > .05). In contrast, greater family NA synchrony had inverse effects, with significant associations with lower adolescent Positive emotions (*b* = −0.44, *SE* = 0.18, 95% CI [–0.81, –0.07], *p* = .028), Engagement (*b* = −0.45, *SE* = 0.13, 95% CI [–0.72, –0.17], *p* = .002), Relationships (*b* = −0.43, *SE* = 0.16, 95% CI [–0.76, –0.10], *t* = −2.61, *p* = .013), and Meaning (*b* = −0.36, *SE* = 0.17, 95% CI [–0.70, –0.02], *p* = .040), wi*t*h no association for accomplishment (*p*s > .05).

**Table 4:** Modeling results for predicting person-level adolescent outcomes.

		Adolescent Outcomes (Person-Level)									
		**Positive Emotions**		**Engagement**		**Relationships**		**Meaning**		**Accomplishment**	
		*b* (SE)	*t*	*b* (SE)	*t*	*b* (SE)	*t*	*b* (SE)	*t*	*b* (SE)	*t*
Coefficients											
	Intercept	6.63 (0.29)	23.18***	7.20 (0.21)	34.16***	7.12 (0.26)	27.84***	6.12 (0.27)	25.18***	6.54 (0.20)	32.17***
	PA Sync	0.37 (0.16)	2.30*	0.34 (0.12)	2.89**	0.33 (0.14)	2.29*	0.28 (0.15)	1.89	0.18 (0.11)	1.61
	NA Sync	–0.44 (0.18)	–2.42*	–0.45 (0.13)	–3.31**	–0.43 (0.16)	–2.61*	–0.36 (0.17)	–2.13*	–0.18 (0.13)	–1.36
Model Fit											
	*F* (2, 36)	3.02*		5.47**		3.41*		2.27		1.29	
	R^2^	.14		.23		.16		.12		.07	
	Adj. R^2^	.10		.19		.11		.06		.01	
		**Perceived Stress**		**Depression**		**Anxiety**		**Stress (DASS-21)**			
		*b* (SE)	*t*	*b* (SE)	*t*	*b* (SE)	*t*	*b* (SE)	*t*		
Coefficients											
	Intercept	2.99 (0.09)	34.58***	0.79 (0.11)	7.37***	0.66 (0.07)	9.23***	0.89 (0.10)	8.77***		
	PA Sync	–0.12 (0.05)	–2.59*	–0.12 (0.06)	–2.03*	–0.01 (0.04)	–0.14	–0.07 (0.06)	–1.26		
	NA Sync	0.13 (0.06)	2.43*	0.14 (0.07)	2.08*	–0.02 (0.05)	–0.37	0.04 (0.06)	0.62		
Model Fit											
	*F* (2, 36)	3.44*		2.73		0.46		1.21			
	R^2^	.16		.12		.03		.06			
	Adj. R^2^	.11		.06		.00		.01			

Level 2 predictors of Overall Family Positive Affect and Negative Affect Synchrony (summed weighted topological overlap values) were grand-mean centered.

*p < .05, **p < .01, ***p < .001.

For maladaptive outcomes, greater family PA synchrony was associated with lower adolescent perceived stress (*b* = –0.13, *SE* = 0.05, 95% CI [–0.22, –0.03], *p* = .014) and depressive symptoms (*b* = –0.12, *SE* = 0.06, CI [–0.24, –0.01], *p* = .049). On the other hand, greater family NA synchrony was associated with higher perceived stress (*b* = 0.13, *SE* = 0.06, CI [0.02, 0.25], *p* = .021) and depressive symptoms (*b* = 0.14, *SE* = 0.07, CI [0.01, 0.28], *p* = .045). Associations with anxiety and DASS-21 stress were not significant (*p*s > .05). Overall, greater family PA synchrony was associated with more favorable adolescent well-being and lower mental health distress, whereas greater family NA synchrony was associated with poorer well-being and elevated distress.

Next, we added adolescents’ overall trait positive affect (PA) and negative affect (NA) as covariates to examine whether associations between family affect synchrony and adolescent well-being remained after accounting for adolescents’ general affect levels (see Table 5). After accounting for trait PA and NA, most previously significant associations between family affect synchrony and positive well-being outcomes were attenuated and no longer statistically significant. Greater family NA synchrony remained significantly associated with lower adolescent Engagement (*b* = −0.30, *SE* = 0.13, 95% CI [–0.57, –0.04], *p* = .027), whereas associations of PA and NA synchrony with Positive emotions, Relationships, Meaning, and Accomplishment were not significant (*p*s > .05). Trait PA was positively associated with Positive emotions (*b* = 1.53, *SE* = 0.28, 95% CI [0.95, 2.10], *p* < .001), Engagement (*b* = 0.87, *SE* = 0.27, 95% CI [0.87, 0.27] *p* = .002), and Meaning (*b* = 1.02, *SE* = 0.30, 95% CI [0.41, 1.64] *p* = .002). Trait NA was not significantly associated with any of the PERMA domains (*p*s > .05).

**Table 5:** Results for predicting person-level adolescent outcomes, accounting for overall affect levels.

		Adolescent Outcomes (Person-Level)									
		**Positive Emotions**		**Engagement**		**Relationships**		**Meaning**		**Accomplishment**	
		*b* (SE)	*t*	*b* (SE)	*t*	*b* (SE)	*t*	*b* (SE)	*t*	*b* (SE)	*t*
Coefficients											
	Intercept	6.75 (0.20)	34.04***	7.24 (0.19)	38.25***	7.19 (0.24)	29.91***	6.74 (0.21)	31.91***	6.64 (0.18)	36.68***
	PA Sync	0.08 (0.12)	0.61	0.21 (0.12)	1.79	0.14 (0.15)	0.34	0.05 (0.13)	0.38	0.07 (0.11)	0.62
	NA Sync	–0.13 (0.14)	–0.92	–0.30 (0.13)	–2.31*	–0.24 (0.17)	–1.44	–0.11 (0.15)	–0.74	–0.05 (0.13)	–0.37
	Adol. Trait PA	1.53 (0.28)	5.38***	0.87 (0.27)	3.28**	0.64 (0.34)	1.87	1.02 (0.30)	3.39**	0.27 (0.26)	1.04
	Adol. Trait NA	–0.30 (0.26)	–1.13	0.01 (0.25)	0.01	–0.40 (0.32)	–1.26	–0.29 (0.28)	–1.04	–0.22 (0.24)	–0.92
Model Fit											
	*F* (4, 33)	10.54***		5.77**		3.05*		4.65**		1.15	
	R^2^	.56		.41		.27		.36		.12	
	Adj. R^2^	.51		.34		.18		.28		.02	
		**Perceived Stress**		**Depression**		**Anxiety**		**Stress (DASS-21)**			
		*b* (SE)	*t*	*b* (SE)	*t*	*b* (SE)	*t*	*b* (SE)	*t*		
Coefficients											
	Intercept	2.99 (0.06)	51.29***	0.79 (0.07)	10.98***	0.08 (0.32)	0.24	0.89 (0.06)	13.85***		
	PA Sync	–0.02 (0.04)	–0.45	0.02 (0.05)	0.41	0.07 (0.04)	2.14*	0.05 (0.04)	1.25		
	NA Sync	0.04 (0.04)	1.03	0.02 (0.05)	0.36	–0.08 (0.04)	–2.21*	–0.06 (0.04)	–1.35		
	Adol. Trait PA	–0.15 (0.08)	–1.85	–0.37 (0.10)	–3.64***	–0.07 (0.08)	0.92	–0.04 (.09)	–0.43		
	Adol. Trait NA	0.47 (0.08)	6.06***	0.49 (0.10)	5.10***	0.37 (0.07)	5.09***	0.62 (0.09)	7.26***		
Model Fit											
	*F* (4, 33)	14.81***		13.87***		5.76**		15.22***			
	R^2^	.64		.63		.48		.65			
	Adj. R^2^	.60		.58		.41		.61			

Level 2 predictors of Overall Family Positive Affect and Negative Affect Synchrony (summed weighted topological overlap values) and Level 2 covariates of adolescent overall positive affect and negative affect (Adol. Trait PA and Adol. Trait NA, respectively) were grand-mean centered.

*p < .05, **p < .01, ***p < .001.

For maladaptive outcomes, the previously observed associations of PA and NA synchrony with perceived stress and depressive symptoms were no longer significant after accounting for adolescents’ trait affect. Trait NA was positively associated with perceived stress (*b* = 0.47, *SE* = 0.08, 95% CI [0.31, 0.63], *p* < .001), depression (*b* = 0.49, *SE* = 0.10, 95% CI [0.29, 0.68], *p* < .001), anxiety (*b* = 0.37, *SE* = 0.07, 95% CI [0.22, 0.51], *p* < .001), and DASS-21 stress (*b* = 0.62, *SE* = 0.09, 95% CI [0.62, 0.09], *p* < .001). Trait PA was associated with lower depressive symptoms (*b* = −0.37, *SE* = 0.10, 95% CI [–0.58, –0.16], *p* < .001). Unexpectedly, when accounting for adolescents’ general affect levels, greater family PA synchrony was associated with higher anxiety (*b* = 0.07, *SE* = 0.04, 95% CI [0.01, 0.14], *p* = .040), whereas greater family NA synchrony was associated with lower anxiety (*b* = −0.08, *SE* = 0.04, 95% CI [–0.16, –0.01], *p* = .034). Because neither synchrony measure was significantly associated with anxiety in the models without trait affect covariates, these findings should be interpreted cautiously.

### Associations with momentary adolescent health and well-being outcomes

As shown in Table 6, within-family (momentary; Level 1) and between-family (overall; Level 2) affect synchrony demonstrated differential associations with momentary adolescent well-being. Momentary (within-family) positive affect (PA) synchrony was largely unrelated to adolescent outcomes, with the exception of a negative association with momentary Relationships (*b* = −2.55, *SE* = 1.24, 95% CI [–4.97, –0.12], *p* = . 040). In contrast, momentary negative affect (NA) synchrony was significantly associated with multiple outcomes, including higher Positive emotions (*b* = 16.24, *SE* = 1.64, 95% CI [13.02, 19.45], *p* < .001), Engagement (*b* = 6.09, *SE* = 2.27, CI [1.64, 10.54], *p* <  = .007), Relationships (*b* = 8.94, *SE* = 1.35, 95%CI [6.28, 11.59], *p* < .001), and Meaning (*b* = 6.82, *SE* = 1.45, 95% CI [3.97, 9.66], *p* < .001), as well as lower momentary stress (*b* = −21.05, *SE* = 2.38, 95% CI [–25.72, –16.38], *p* < .001). Associations with accomplishment were not significant.

**Table 6:** Summary of multilevel modeling results for predicting momentary adolescent outcomes.

		Adolescent Outcomes (Momentary-Level)											
		***m*Positive Emotions**		***m*Engagement**		***m*Relationships**		***m*Meaning**		***m*Accomplishment**		**Momentary Stress**	
		*b* (SE)	*t*	*b* (SE)	*t*	*b* (SE)	*t*	*b* (SE)	*t*	*b* (SE)	*t*	*b* (SE)	*t*
Fixed Effects													
	Intercept	67.03 (2.06)	36.62***	56.67 (2.60)	21.89***	71.69 (1.95)	36.87***	65.51 (3.01)	21.77***	58.32 (3.15)	18.49***	32.81 (2.81)	11.70***
	Moment PA Sync	–0.52 (1.50)	–0.35	–2.62 (2.07)	–1.26	–2.55 (1.24)	–2.06*	0.39 (1.33)	0.29	0.52 (1.93)	0.27	–3.09 (1.18)	–1.42
	Moment NA Sync	16.24 (1.64)	9.91***	6.09 (2.27)	2.68**	8.94 (1.35)	6.60***	6.82 (1.45)	4.70***	0.45 (2.11)	0.21	–21.05 (2.38)	–8.84***
	Overall PA Sync	3.44 (1.17)	2.95**	0.75 (1.48)	0.51	3.51 (1.11)	3.18**	3.52 (1.71)	2.06*	0.44 (1.79)	0.25	–2.73 (1.60)	–1.71
	Overall NA Sync	–2.66 (1.33)	–2.00	–0.19 (1.67)	–0.12	–2.97 (1.26)	–2.37*	–3.23 (1.95)	–1.66	1.46 (2.04)	0.72	4.40 (1.82)	2.42*
Random Effects													
	Intercept Variance (SD)	157.30 (12.54)		247.50 (15.73)		143.30 (11.97)		353.30 (18.80)		378.70 (19.46)		291.20 (17.07)	
	Residual (SD)	211.70 (14.55)		406.20 (20.16)		144.40 (12.02)		165.90 (12.88)		351.80 (18.76)		447.20 (21.15)	
Model Fit													
	AIC, BIC	6262, 7314		6763, 6796		6005, 6038		6137, 6169		6675, 6708		6282, 6314	
	ICC	.43		.38		.50		.68		.52		.43	
	Conditional R^2^	.52		.39		.57		.71		.54		.52	
	Marginal R^2^	.16		.01		.15		.08		.05		.16	

Level 1 predictors of Momentary Family PA and NA Synchrony were person-centered. Level 2 predictors of Overall Family PA and NA Synchrony (using summed weighted topological overlap values) were grand-mean centered.

*p < .05, **p < .01, ***p < .001.

At the between-family level, greater overall PA synchrony was associated with higher momentary positive emotions (*b* = 3.44, *SE* = 1.17, 95% CI [1.19, 5.70], *p* = .006), relationships (*b* = 3.51, *SE* = 1.11, 95% CI [1.38, 5.64], *p* = .003), and meaning (*b* = 3.52, *SE* = 1.71, 95% CI [0.22, 6.82], *p* = .047), with no significant associations for engagement, accomplishment, or stress (*p*s > .05). Greater overall NA synchrony was associated with lower momentary relationships (*b* = −2.97, *SE* = 1.26, 95% CI [–5.40, –0.55], *p* = .023) and higher momentary stress (*b* = 4.40, *SE* = 1.82, 95% CI [0.90, 7.91], *p* = .020), with no other significant associations (*p*s > .05).

Lastly, we added adolescents’ person-centered momentary PA and NA levels to the models to examine whether associations between family affect synchrony and momentary adolescent well-being remained after accounting for adolescents’ own momentary affective states (see Table 7 for details). After accounting for momentary adolescent PA and NA, the previously observed associations between momentary family NA synchrony and Positive emotions, Engagement, Relationships, Meaning, and stress were no longer statistically significant (*p*s > .05). In contrast, the negative association between momentary family PA synchrony and Relationships remained significant (*b* = −2.47, *SE* = 1.11, 95% CI [–4.64, –0.31], *p* = .026). Thus, most within-family synchrony effects were attenuated after accounting for whether adolescents were experiencing higher or lower PA and NA than was typical for them at a given occasion.

**Table 7:** Summary of multilevel modeling results for predicting momentary adolescent outcomes, accounting for overall affect levels.

		Adolescent Outcomes (Momentary-Level)											
		***m*Positive Emotions**		***m*Engagement**		***m*Relationships**		***m*Meaning**		***m*Accomplishment**		**Momentary Stress**	
		*b* (SE)	*t*	*b* (SE)	*t*	*b* (SE)	*t*	*b* (SE)	*t*	*b* (SE)	*t*	*b* (SE)	*t*
Fixed Effects													
	Intercept	67.01 (2.06)	32.56***	56.86 (39.92)	21.90***	71.68 (1.95)	34.85***	65.51 (3.01)	36.99***	58.33 (3.15)	18.49***	32.89 (2.81)	11.70***
	Moment PA Sync	–0.48 (1.20)	–0.40	–2.75 (1.98)	–1.40	–2.47 (1.11)	–2.24*	0.33 (1.24)	0.27	0.47 (1.89)	0.25	–3.45 (1.96)	–1.76
	Moment NA Sync	1.76 (1.67)	1.05	–0.37 (2.75)	–0.14	–0.94 (1.54)	–0.61	0.81 (1.72)	0.47	–4.32 (2.63)	–1.64	–0.53 (2.72)	–0.19
	Overall PA Sync	3.46 (1.17)	2.96**	0.76 (1.48)	0.51	3.51 (1.11)	3.18**	3.52 (1.71)	2.06*	0.44 (1.79)	0.25	–2.74 (1.60)	–1.71
	Overall NA Sync	–2.68 (1.33	–2.02	–0.20 (1.68)	–0.12	–2.98 (1.26)	–2.37*	–3.23 (1.95)	–1.66	1.46 (2.04)	0.72	4.41 (1.82)	2.43*
	Moment Adol. PA	12.18 (0.75)	16.34***	10.03 (1.23)	8.18***	6.99 (0.69)	10.20***	7.43 (0.77)	9.70***	5.89 (1.18)	5.02***	–8.07 (1.22)	–6.65***
	Moment Adol. NA	8.38 (1.50)	–5.60***	0.76 (2.47)	0.31	–7.00 (1.38)	–5.08***	–1.16 (1.54)	–0.75	–0.93 (2.36)	–0.40	20.87 (2.44)	8.56***
Random Effects													
	Intercept Variance (SD)	161.90 (12.72)		249.40 (15.79)		145.00 (12.04)		354.40 (18.83)		379.40 (1.48)			
	Residual (SD)	136.10 (11.67)		369.50 (19.22)		115.40 (10.74)		144.00 (12.00)		338.70 (18.40)			
Model Fit													
	AIC, BIC	5966, 6008		6692, 6734		5844, 5886		6034, 6075		6645, 6686			
	ICC	.54		.40		.56		.71		.53			
	Conditional R^2^	.69		.44		.66		.74		.56			
	Marginal R^2^	.32		.07		.23		.12		.06			

Level 1 predictors of momentary family PA and NA synchrony were person-centered (Moment PA Sync and Moment NA Sync, respectively). Level 2 predictors of Overall Family PA and NA Synchrony (using summed weighted topological overlap values) were grand-mean centered. Level 1 covariates of adolescent momentary PA and NA (Moment Adol. PA and Moment Adol. NA, respectively) were calculated as the mean of the respective PA and NA items at each measurement occasion and were person-mean centered; thus, these covariates represent each adolescent’s own average level of PA and NA for that moment.

*p < .05, **p < .01, ***p < .001.

At the between-family level, the pattern of associations remained largely unchanged. Greater overall family PA synchrony remained significantly associated with higher momentary Positive emotions (*b* = 3.46, *SE* = 1.17, 95% CI [1.20, 5.71], *p* = .005), Relationships (*b* = 3.51, *SE* = 1.11, 95% CI [1.38, 5.65], *p* = .003), and Meaning (*b* = 3.52, *SE* = 1.71, 95% CI [.02, 6.83], *p* = .046). Greater overall family NA synchrony also remained significantly associated with lower momentary Relationships (*b* = −2.98, *SE* = 1.26, 95% CI[–5.41, –0.55], *p* = .023) and higher momentary stress (*b* = 4.41, *SE* = 1.82, 95% CI[0.91, 7.92], *p* = .020). These findings indicate that the between-family synchrony associations were robust to adolescents’ occasion-specific deviations in PA and NA.

## Discussion

The current study extends research on family emotion dynamics by conceptualizing emotional synchrony as a family-level, triadic process. Rather than treating synchrony as a property of isolated dyads or laboratory-based interactions, we examined emotional synchrony as an experience that unfolds in the flow of daily family life. This approach aligns with family-level perspectives on emotion regulation and coregulation [35] and responds to calls to study parent-adolescent emotion processes and relationships across multiple time-scales [13,34]. Using EMA and dynamic network modeling, we examined how adolescents, mothers, and fathers were emotionally linked across a one week period. We also tested whether those emotional synchrony patterns were associated with adolescents’ momentary and overall well-being. In doing so, this study builds on prior work showing that families are best understood as interdependent systems rather than as the sum of separate relationships [25,29,60]. More broadly, it advances a more ecologically valid account of how family emotional life may shape adolescent adjustment in real time.

Overall, our findings suggest that family emotional synchrony is not a fixed or uniform feature of family life, but a variable property of the family emotional system. This framing aligns with family systems perspectives that treat family relational processes as dynamic and adaptive rather than fixed [19], and with evidence that individual families differ substantially in how their relational processes unfold [60]. Families differed meaningfully in both positive and negative affect synchrony, with positive affect synchrony generally exceeding negative affect synchrony across the sample. At the same time, both forms of synchrony mattered for adolescents’ mental health. This is consistent with theoretical models emphasizing the role of the broader family emotional climate in shaping children’s emotion regulation and adjustment [4]. Family emotional synchrony was significantly associated with adolescent well-being outcomes at both the person-level and the momentary level. This convergence across levels underscores the value of examining family emotional processes across multiple timescales [34]. These findings indicate that synchrony is relevant not only as a broader family characteristic, but also as an ongoing predictor of adolescents’ lived emotional context. This is consistent with evidence that day-to-day fluctuations in family relational processes are linked to adolescents’ daily well-being [5]. Rather than treating family emotions as background noise, these findings point to synchrony as a meaningful relational process through which family life may shape adolescent well-being from moment to moment and across days.

### Temporal patterns of family emotional synchrony

One of the clearest patterns in the data was that positive affect synchrony was higher on average than negative affect synchrony across families. This finding suggests that positive affective states may be more readily coordinated across everyday family life than shared negative emotions. Because the present study operationalized synchrony as overlap in how affective states changed together over time within family dynamic networks, this finding is best interpreted as a difference in family-level coordination, rather than simply a difference in how often positive versus negative emotions occurred. This pattern fits interpersonal views of emotion that treat emotion as something that unfolds not only within persons but also between them [16], and with family-level coregulation perspectives that emphasize that emotion processes unfold across multiple family subsystems rather than only within isolated dyads [35]. In family life specifically, positive emotion may be easier to sustain through everyday reciprocal interaction, as children’s positive emotion has been shown to persist when parents or siblings join with positive emotion, touch, and shared activity [61]. This interpretation is also broadly consistent with triadic family work showing that families differ in the extent to which they occupy synchronous affective states across interaction [12]. By contrast, lower synchrony in negative affect may indicate that negative emotional states are more differentiated across family members, or that parent–adolescent connectedness can buffer the day-to-day spread of negative emotion rather than allowing it to become a fully shared family state [62].

The triad family network structures reinforce this interpretation. Negative affect networks were more often ‘disconnected’ (i.e., no associations among any of the family members) and less often ‘closed’ (i.e., high associations between all family members) than positive affect networks, suggesting that negative emotion may be more unevenly distributed across family members or remain localized within particular dyads rather than spreading across the whole triad. This pattern may reflect adaptive family processes: in healthy families, negative emotions may be more individually contained, partly because adolescents are developmentally motivated toward emotional autonomy from parents [4]. Parents may also engage in deliberate emotion regulation, buffering negative states from the family system, particularly in the presence of adolescent children [3,4].

At the same time, the substantial variability across families is itself meaningful: some triads showed strong synchrony across both positive and negative affect, whereas others showed little synchrony at all. This heterogeneity suggests that there is no single emotional structure that characterizes family life; instead, families appear to differ in how tightly connected their emotional systems are and in whether that connectedness is expressed similarly across positive and negative emotions. In this sense, the present findings extend earlier triadic work showing that families differ in the extent to which they occupy synchronous affective states [12] and daily-life network work showing that family affect systems can be modeled as distinct, family-specific structures [25]. A network perspective is especially useful here because it shifts attention away from any single tie in isolation and toward the configuration of the family system as a whole [29,60].

### Family emotional synchrony and overall adolescent well-being

At the broader family level, emotional synchrony across the week was not merely a descriptive feature of family life, but was systematically related to adolescents’ overall well-being. The results indicated that higher positive affect (PA) synchrony was associated with adolescents’ positive emotions, engagement, and relationships, whereas higher negative affect (NA) synchrony was associated with lower positive emotions, engagement, relationships, and meaning. In addition, higher PA synchrony was linked to lower perceived stress and lower depressive symptoms, whereas higher NA synchrony was related to higher perceived stress and higher depressive symptoms. These findings suggest that the overall emotional synchrony and coordination of the family is meaningfully linked to adolescents’ broader psychological functioning, and that the valence of that synchrony matters. A family system characterized by coordinated positive emotion may provide a more emotionally supportive context for adolescents’ overall psychological well-being and mental health, whereas coordinated negative emotion may reflect a more burdensome family emotional climate [2–4,12].

One implication of the PA synchrony findings is that positive family synchrony may be especially relevant to the hedonic and relational dimensions of adolescent well-being. The clearest associations emerged for positive emotions, engagement, and relationships, but not for meaning or accomplishment. This pattern suggests that when families are more coordinated in positive emotional experience, adolescents may be more likely to feel emotionally uplifted, socially connected, and actively engaged in daily life, without necessarily differing in more reflective or achievement-oriented domains. This interpretation is consistent with evidence that stronger parent–adolescent connectedness is associated with adolescents’ subjective well-being and eudaimonic functioning [5], and that day-to-day parent–adolescent closeness is associated with greater life satisfaction, whereas conflict is associated with lower life satisfaction [63]. It is also in line with work suggesting that well-being is a dynamic, multidimensional, and partly person-specific system, such that some domains may be more strongly linked to family processes than others [30]. In this sense, PA synchrony may be especially relevant to the more emotional and interpersonal aspects of well-being, rather than to all PERMA domains equally.

The pattern for NA synchrony was broader and, in some ways, more consequential. Higher NA synchrony was associated not only with lower positive emotions, engagement, and relationships, but also with lower meaning, suggesting that synchrony in negative emotion at the family level may signal a more pervasive and chronically stressful family emotional climate. This interpretation fits the tripartite model of family influence on emotion regulation, which proposes that children and adolescents are shaped not only by parents’ direct responses to emotion, but also by modeling processes and by the broader emotional climate of the family [4]. It also aligns with Fosco and Grych’s [3] family-systems work showing that family emotional climate, interparental conflict, and parent warmth are jointly relevant to youths’ emotion regulation development. More generally, this is compatible with evidence that adolescents’ well-being depends not simply on the presence of one supportive relationship, but on how the family network as a whole is organized [29]. From this perspective, when NA becomes coordinated across family members over time, adolescents may be living in an environment in which distress is more mutually reinforced, harder to contain, and more likely to generalize into broader psychological functioning [3,4].

The associations with stress and depressive symptoms further support this interpretation. Adolescents from families with higher PA synchrony reported lower perceived stress and fewer depressive symptoms, whereas adolescents from families with higher NA synchrony reported greater stress and more depressive symptoms. These findings extend prior work linking family affect dynamics to adolescent depression. For example, Hollenstein et al. [12] found that families of depressed adolescents spent less time in synchronous affective states, and Bodner et al. [2] showed that families of depressed adolescents displayed more maladaptive affective dynamics during family interaction. The present findings build on that literature by suggesting a graded family-level pattern in daily life: the degree and valence of family emotional synchrony may be associated with adolescents’ stress and depressive symptoms along a continuum. This interpretation is also consistent with work showing that supportive parent–adolescent relationships are linked to better adjustment and fewer depressive symptoms, in part through emotion regulation processes [32].

The non-significant findings are informative as well. Accomplishment was not significantly predicted by either PA or NA synchrony, which may suggest that achievement-related well-being depends more strongly on school demands, peer comparison, self-regulation, and personal goals than on the family’s general emotional coordination. Anxiety also was not significantly associated with overall family synchrony. One possible interpretation is that anxiety may be less tied to the family’s overall emotional valence and more tied to more specific family processes such as parental control, overprotection, and intrusiveness, which have been linked to child and adolescent anxiety in both meta-analytic and more recent work [1,64]. More broadly, these null findings reinforce the idea that not all aspects of well-being are equally sensitive to the same family processes. Altogether, the present person-level results suggest that family emotional synchrony is especially relevant to adolescents’ emotional, relational, and stress-related functioning, while other domains may depend on more specific mechanisms or broader developmental contexts [1].

### Family emotional synchrony and momentary adolescent well-being

Whereas the person-level analyses examined whether adolescents from more emotional synchronized families reported better overall well-being, the momentary analyses addressed a different question: on occasions when a family was more synchronized than usual, did adolescents report feeling better or worse at that same moment? We also controlled for the overall family synchrony to ensure the association between momentary family synchrony and concurrent adolescent momentary well-being, is reflected above and beyond broader between-family differences in overall synchrony.

The most striking finding was that momentary family NA synchrony was positively associated with several dimensions of concurrent adolescent momentary well-being and negatively associated with adolescent momentary stress. In moments when families were more synchronized in negative affect than was typical for them, adolescents reported higher positive emotions, engagement, relationships, and meaning, as well as lower stress. Although this pattern may initially seem counterintuitive, especially given that higher overall NA synchrony at the person level was associated with poorer adolescent well-being and greater distress, one interpretation is that momentary NA synchrony may sometimes reflect emotional attunement during difficult moments rather than a globally negative family climate. When family members are aligned in negative emotion at a given occasion, adolescents may experience that shared affect as a sign that their distress is being recognized and not carried alone. In that short-term context, shared negative affect may coincide with greater connection, validation, or coordinated coping. This interpretation is consistent with the social sharing of emotion literature, which emphasizes that emotional experiences often elicit interpersonal sharing and can serve affiliative as well as regulatory functions [15]. It is also compatible with work suggesting that synchrony is not inherently beneficial or harmful, but depends on context, timing, and flexibility [65]. By contrast, higher overall NA synchrony likely indexes a more stable family pattern in which negative affect is repeatedly shared across time, reflecting a more burdensome family emotional climate in which distress becomes recurrent, mutually reinforced, and more difficult to regulate rather than a single moment of attunement [3,4]. In this sense, shared negative affect may be beneficial in social-connecting in the short term, but maladaptive when it becomes a stable family pattern over time.

The findings for momentary PA synchrony were notably different. Occasion-level increases in PA synchrony were largely unrelated to adolescents’ concurrent momentary outcomes, with the exception of a negative association with momentary relationships. Because this was the only significant momentary PA effect, and because it ran counter to the broader pattern, it should be interpreted cautiously. One possibility is that transient increases in shared positive affect do not necessarily translate into adolescents’ immediate sense of relational closeness, especially if those moments reflect diffuse group positivity rather than support specifically directed toward the adolescent. More generally, the momentary level results suggest that positive synchrony may not operate as strongly through acute occasion-level alignment as negative synchrony does. Two potentially complementary explanations may help account for this pattern. Because positive affect is normatively more common and represents something closer to a default affective state in daily life, momentary increases in shared positive affect may be less distinctive against an already elevated baseline, making it harder for occasion-level PA synchrony to register as a salient event with its own predictive value [66]. Negative affect, by contrast, occurs less often, and its shared experience may typically call for more active engagement or coordinated regulation among family members, which could make moments of shared negative affect more psychologically salient and consequential for adolescents’ concurrent well-being than comparable moments of shared positive affect [67]. This interpretation fits the broader pattern in the present findings, where overall PA synchrony, rather than momentary PA synchrony, was more consistently associated with adolescents’ well-being, and is also consistent with daily family research indicating that more stable parent–adolescent connectedness and closeness are linked to adolescents’ well-being and life satisfaction [5,63].

Overall, these findings highlight the value of ecological momentary assessment for identifying micro-level family processes that would likely be obscured in global reports alone. By separating occasion-level synchrony within families from broader between-family differences in synchrony, the analyses show that the same emotional process can carry different implications depending on how it is modeled and at what timescale it is examined [34]. In the present study, momentary shared negative affect appeared to function as a marker of short-term emotional attunement, even though higher overall negative synchrony was associated with poorer broader functioning. That distinction may be one of the more important contributions of the findings.

### Synchrony effects beyond adolescents’ general affect levels

To evaluate whether family synchrony predicted adolescent outcomes beyond adolescents’ own typical or concurrent affect, we re-estimated the person-level and momentary models with adolescents’ general affect levels included as covariates. At the person level, most associations between family synchrony and adolescent well-being were attenuated to nonsignificance once adolescents’ average trait positive and negative affect were included, including the associations with positive emotions, relationships, meaning, perceived stress, and depressive symptoms. The one exception was negative affect synchrony’s association with lower engagement, which remained significant. Engagement reflects attentional and behavioral absorption rather than a purely affective state [68], which may explain why it retained a unique association with family synchrony that was not redundant with adolescents’ general affective tendencies.

As expected, trait positive and negative affect were themselves associated with several outcomes, consistent with the tripartite model of anxiety and depression, in which negative affectivity functions as a shared vulnerability across anxiety and depressive symptoms while low positive affect is more specific to depression [69]. However, an unexpected finding emerged for anxiety: once trait affect was included, greater family PA synchrony became associated with higher adolescent anxiety, while greater family NA synchrony became associated with lower anxiety. This might be due to a statistical suppression effect, in which a covariate correlated with the predictor can reveal or reverse an association that was otherwise masked [70], and should be interpreted cautiously given its modest magnitude.

At the momentary level, within-family associations showed a similar pattern of attenuation once adolescents’ own average momentary affect was included, with the exception of momentary positive affect synchrony, which continued to predict lower momentary relationships. This may indicate that especially tight moment-to-moment PA synchrony can, in some instances, reflect an enmeshed or overly reactive family dynamic rather than adaptive emotional coordination, consistent with work suggesting that the benefits of interpersonal synchrony depend on context, timing, and flexibility rather than magnitude alone [65]. Between-family synchrony, by contrast, remained largely robust to this adjustment, continuing to predict adolescents’ momentary well-being and stress beyond their concurrent affect, reinforcing the value of separating within-family and between-family timescales [34].

### Theoretical contributions

The present study makes several theoretical contributions to family research. First, it advances family systems theory by operationalizing the family as a dynamic emotional network rather than as a collection of separate dyads. Family systems perspectives have long emphasized that family members are interdependent and that family functioning cannot be reduced to isolated pairwise ties [35]. The current findings extend that perspective by showing that emotional synchrony can be modeled as a triadic, family-level process that unfolds across adolescents, mothers, and fathers simultaneously in daily life. In doing so, the study builds on recent family-network research showing that family processes are often family-specific and better understood at the level of the broader relational configuration rather than any single tie in isolation [25,29,60].

Second, the study makes a methodological contribution by applying dynamic exploratory graph analysis (dynEGA) to EMA data from all three family members. DynEGA is designed to estimate dynamic structure in multivariate time series across multiple timescales and levels of analysis, making it especially well suited to modeling emotional processes that are both time-varying and relational [9,40]. In the present study, this approach made it possible to capture the temporal co-variation of emotions across the full family triad, which is a substantial advance over cross-sectional designs, single-informant reports, or dyadic-only approaches. More broadly, the study responds to calls to examine parent–adolescent emotion processes across multiple timescales and in ecologically valid contexts [8,13].

Third, the study demonstrates the value of combining EMA with multilevel dynamic network modeling to distinguish family emotional processes that operate at different timescales. By separating momentary within-family synchrony from broader between-family synchrony, the analyses showed that the same emotional process (i.e., negative affect synchrony) can carry different implications depending on how it is modeled. This is an important contribution because such divergence would likely be obscured in cross-sectional, global-report, or dyadic designs. In this sense, the study not only identifies a novel pattern in family emotional dynamics, but also shows that timescale is itself a critical feature of family emotional synchrony [8,13,34].

### Limitations and future directions

Several limitations should be considered when interpreting these findings. The sample was relatively small (*N* = 40 families) and demographically narrow, consisting primarily of high-SES, two-parent, cohabiting households, which limits generalizability to more diverse family structures (e.g., non-cohabiting or separated families) and socioeconomic contexts. In addition, the study covered only one week of daily life, which may not capture longer-term developmental, seasonal, or contextual variation in family synchrony. The analyses also relied on self-reported affect, which may be influenced by reporting biases, including social desirability and shared method variance. Further, the study was correlational, so the observed associations should not be interpreted causally; bidirectional processes are plausible, such that adolescent well-being may also shape family emotional synchrony. Finally, the use of statistical jitter for zero-variance variables should be noted as additional limitations, even though the analytic decisions were transparent and methodologically justified. Relatedly, the mPERMA Positive emotion element shares conceptual content with the SAS positive affect items used to construct PA synchrony, raising the possibility of shared method variance. This concern is addressed more directly in additional analyses, which showed that most person-level PA and NA synchrony associations were substantially attenuated once adolescents’ trait affect was included as a covariate, suggesting that shared method variance, rather than item overlap alone, may account for a meaningful portion of these effects. The between-family momentary associations, by contrast, remained robust to this adjustment, suggesting they are less susceptible to this concern.

Future work should extend this design in several ways. Longer studies spanning months or years would help clarify how family synchrony develops over time and whether it predicts later adjustment. Replication in more diverse samples, including single-parent families, stepfamilies, and lower-SES households, is also essential. Extending this work to racially, ethnically, and culturally diverse families, including immigrant families, will also be important, given evidence that experiences such as discrimination can shape well-being in ways that are not fully buffered by social support [71]. It will also be important to test moderators such as adolescent age, gender, attachment security, and family conflict, given evidence that parent–adolescent emotional dynamics vary across developmental and relational contexts [13,60]. Another promising direction is the integration of physiological synchrony measures, such as cortisol or autonomic activity, alongside self-reported affect, as prior work suggests that physiological linkage can reveal family coordination processes not captured by self-report alone [72,73]. More broadly, the present findings raise the possibility that family emotional synchrony could become a meaningful target for family-based prevention or intervention efforts aimed at improving emotion regulation and adolescent adjustment, though that possibility remains to be tested directly [32,35].

### Conclusion

In conclusion, the present study highlights family emotional synchrony as a clinically and developmentally meaningful systems-level construct. By modeling adolescents, mothers, and fathers as part of a single dynamic emotional network, the findings show that synchrony is not simply a dyadic phenomenon, but a property of the family system that relates to adolescent well-being at both broader and momentary levels, with the broader, family-level pattern of synchrony proving especially robust to adolescents’ own general and momentary affect. The study also demonstrates the value of combining ecological momentary assessment with dynamic network methods to capture how family emotional processes unfold in everyday life, where they are likely to matter most. Taken together, these findings suggest that family emotional synchrony is an important mechanism through which the family’s emotional climate may shape adolescent adjustment, and they underscore the value of studying families as dynamic, interdependent systems rather than as collections of separate relationships [8,25,35].

## Supporting information

S1 File S1 Appendix. Additional statistical and methodological details.
(DOCX)
