# Supplementary material for: Emotional synchrony in family networks is linked to adolescent well-being in daily life
Source: PLoS One. 2026 Nov 11;21(11):e0362751. doi: 10.1371/journal.pone.0362751 (PMC13754097; doi:10.1371/journal.pone.0362751)
Supplement: Supplementary file 1 [file pone.0362751.s001.docx]

# Supporting Information: S1 Appendix

*Additional Statistical and Methodological Details*

## S1.1 Descriptive Statistics

### Descriptive Statistics of Family Momentary Affect

The descriptive statistics for the 18 Subcomponents of Affect Scale (SAS [1]) are in Table S1 Table 1. For all SAS items, participants were prompted, “Please indicate the extent to which you feel the emotion listed below right now,” and had response options of 1 = *Very slightly/Not at all,* 2 = *A little*, 3 = *Moderately*, 4 = *Quite a bit*, and 5 = *Extremely*.

**S1 Table 1. Descriptive Statistics of Subcomponents of Affect Scale (SAS) for Family Triad Members**

| **Affect Item** | **Family Member** | | |
| --- | --- | --- | --- |
|  | Child | Dad | Mom |
|  | *M*(*SD*) | *M*(*SD*) | *M*(*SD*) |
| Positive Affect Overall | 1.67 (0.93) | 1.83 (0.94) | 1.70 (0.86) |
| Lively | 1.29 (1.19) | 1.53 (1.16) | 1.33 (1.14) |
| Full of pep | 1.00 (1.16) | 1.36 (1.16) | 1.07 (1.10) |
| Energetic | 1.23 (1.21) | 1.53 (1.12) | 1.36 (1.11) |
| Happy | 1.98 (1.23) | 2.01 (1.13) | 2.06 (1.02) |
| Pleased | 1.17 (1.23) | 1.96 (1.12) | 1.93 (1.09) |
| Cheerful | 1.45 (1.22) | 1.68 (1.16) | 1.61 (1.16) |
| At ease | 1.98 (1.26) | 2.09 (1.14) | 1.93 (1.15) |
| Calm | 2.17 (1.24) | 2.20 (1.06) | 2.09 (1.00) |
| Relaxed | 2.13 (1.29) | 2.08 (1.14) | 1.95 (1.13) |
| Negative Affect Overall | 0.39 (0.55) | 0.29 (0.47) | 0.29 (0.51) |
| Sad | 0.39 (0.78) | 0.25 (0.60) | 0.29 (0.67) |
| Depressed | 0.28 (0.67) | 0.23 (0.59) | 0.19 (0.54) |
| Unhappy | 0.45 (0.82) | 0.30 (0.65) | 0.27 (0.66) |
| Nervous | 0.73 (1.05) | 0.36 (0.73) | 0.43 (0.80) |
| On edge | 0.47 (0.86) | 0.44 (0.77) | 0.45 (0.84) |
| Tense | 0.54 (0.90) | 0.48 (0.77) | 0.54 (0.86) |
| Hostile | 0.19 (0.58) | 0.14 (0.47) | 0.10 (0.41) |
| Resentful | 0.26 (0.64) | 0.25 (0.63) | 0.23 (0.69) |
| Angry | 0.22 (0.60) | 0.19 (0.52) | 0.16 (0.57) |

For total EMAs collected, Child *N* = 755, Dad *N* = 796, Mom *N* = 814.

### Descriptive Statistics of Trait- and Momentary-Level Adolescent Outcome Variables

The descriptive statistics for the outcome variables of interest (Trait-Level and Momentary-Level) are in S1 Table 2.

**S1 Table 2. Descriptive Statistics of Momentary and Trait-Level Adolescent Outcomes**

| **Variable** | | ***M* (*SD*)** | **Min** | **Max** |
| --- | --- | --- | --- | --- |
| Trait-Level Outcomes | |  |  |  |
|  | Positive emotions | 6.64 (1.88) | 1.33 | 9.33 |
|  | Engagement | 7.21 (1.46) | 3.67 | 10.00 |
|  | Relationships | 7.13 (1.70) | 2.00 | 10.00 |
|  | Meaning | 6.62 (1.69) | 2.00 | 9.67 |
|  | Accomplishment | 6.62 (1.69) | 2.00 | 8.67 |
|  | Perceived Stress (PSS) | 2.99 (0.57) | 1.60 | 4.30 |
|  | Depression | 0.79 (0.6) | 0.00 | 2.71 |
|  | Anxiety | 0.67 (0.44) | 0.00 | 1.57 |
|  | Stress (DASS-21) | 0.89 (0.63) | 0.00 | 2.29 |
|  | Positive Affect (PANAS) | 3.21 (0.75) | 1.30 | 4.60 |
|  | Negative Affect (PANAS) | 2.22 (0.85) | 1.00 | 4.50 |
| Momentary Level Outcomes | |  |  |  |
|  | mPositive emotions | 67.82 (20.44) | 2.00 | 100.00 |
|  | mEngagement | 57.24 (25.37) | 0.00 | 100.00 |
|  | mRelationships | 72.48 (17.82) | 5.00 | 100.00 |
|  | mMeaning | 66.52 (22.95) | 0.00 | 100.00 |
|  | mAccomplishment | 58.55 (27.50) | 0.00 | 100.00 |
|  | Momentary Stress | 31.86 (28.27) | 0.00 | 100.00 |

*Note.* Perceived Stress derived from the Perceived Stress Scale (PSS), Stress is derived from the Depression, Anxiety, Stress Scale (DASS-21), and Positive and Negative Affect are derived from the Positive and Negative Affect Schedule (PANAS).

## S1.2 Network and Dimension Estimation

Dynamic Exploratory Graph Analysis (DynEGA [2]) proceeds in three estimation stages: (first-order) derivatives, networks, communities. To estimate the derivatives, the Generalized Local Linear Approximation (GLLA [3]) is applied to each variable of each individual’s time series separately. Taking a single variable, , of a single participant where is the length of the time series as an example (e.g., length of 9), a time-delay embedding matrix is created for a given time window (e.g., 5):$X=\left[ X_{1},X_{2},\ldots,X_{t} \right]t$

where the offset of consecutive measurement points is equal to 1. This time-delay embedding matrix is created for each variable for each participant through their number of time points, $t$. Using these time-delay embeddings, a weight matrix, $L$, is created to estimate the derivatives, $Y$, such that:

$$Y=X_{T}L\left( L^{'}L \right)^{-1},$$

where $X_{T}$ is the time-delay embedding for the values in variable $X$, $L$ takes the dimensions $t\times\alpha$ with $t$ representing the number of time points and $alpha$ representing the order of derivatives ranging from 0 to $\alpha$. For this study, $\alpha=1$ or the first-order derivatives were used. $L_{\alpha}$ is defined as:

$$\frac{\left[ \Delta_{t}\left( v-v‾ \right) \right]^{\alpha}}{\alpha!},$$

where $v$ and $v‾$ represent a vector from 1 to the length of the time-delay embedding window (i.e., 5) and the average of that vector, respectively. The end result is a $\left( t-4 \right)\times p$ matrix, $Y$, representing the first-order derivatives of the time series where $p$ is the number of variables for each participant. A sample-level matrix of derivatives is created by stacking all participants’ derivatives row-wise, forming a new matrix with the dimensions $\sum\left( t_{i}-4 \right)\times p$ where $n$ is the number of participants.

These derivatives for the sample-level and each family (each family of three participants had a unique identifier of FamilyID). This was treated as an “individual”-level of analysis to represent individual families (hereafter referred to as the “individual family-level”). The derivatives for the sample-level and each individual family-level are submitted to the graphical least absolute shrinkage and selection operator (GLASSO [4]) with extended Bayesian information criterion (EBIC [5,6]). The GLASSO algorithm uses an optimization method called block coordinate descent to maximize the penalized log-likelihood:

$$logdet\Theta-tr\left( S\Theta\right)-\lambda\parallel\Theta\parallel_{1},$$

where $\Theta$ is the (penalized) inverse covariance matrix, $S$ is the empirical covariance matrix, $tr$ denotes trace, and $\parallel\Theta\parallel_{1}$ is the $\mathcal{l}_{1}$ norm (sum of the absolute values of the elements in $\Theta$). The parameter, $\lambda$, controls the strength of the penalty or the extent to which values of $\Theta$ shrink. For the $\mathcal{l}_{1}$ norm, there is an additional “soft-thresholding” step that sets parameters (elements of $\Theta$) less than $\lambda$ to 0.

To determine $\lambda$, several values (e.g., 100), which are defined by a logarithmic spacing from a maximum value ($max\left| R \right|$ where $R$ is a correlation matrix) to a minimum value ($max\left| R \right|\times0.1$), are used to estimate different networks. These networks vary along from more sparse (i.e., fewer edges and more non-edges; larger $\lambda$) to more dense (i.e., more edges and few non-edges; smaller $\lambda$). To select from these networks, the EBIC is used:

$$EBIC=-2logL+Elog\left( N \right)+4\gamma Elog\left( p \right)$$

and

$$logL=\frac{1}{2}logdet\left( \Theta\right)-tr\left( S\Theta\right)$$

where is the Gaussian log-likelihood and is the number of edges in the network. By default, . The value of that minimizes EBIC is used as the network. Specific to the exploratory graph analysis (EGA [7,8]) framework, decreases by 0.25 when there are disconnected nodes in the network. This step proceeds until there are no disconnected nodes in the network or where the latter case is equivalent to the Bayesian information criterion. The results of the network estimation step are networks, and , at the individual- and sample-level (respectively).$LE\gamma=0.50\lambda\gamma\gamma=0W_{n}W_{s}$

## S1.3 Louvain Algorithm

For each network at the individual- and sample-level, the lower-order Louvain algorithm [9,10]. The Louvain algorithm seeks to identify communities in a network by maximizing the modularity function (),$Q$

$$Q=\frac{1}{2m}\sum\left( W_{ij}-\rho\frac{k_{i}k_{j}}{2m}s_{i}s_{j} \right),$$

where $m$ represents $\sum W$, $k_{i}$ is $\sum W_{i}$ for variable $i$, and $s_{i}s_{j}$ equals 1 when variable $i$ and $j$ belong to the same community and equals 0 when they belong to different communities. The term $\rho=1$ by default.

The Louvain algorithm begins by having all nodes as individual communities and one-by-one merging nodes into the community that produces the greatest increase in . If there are no merges that produce a positive increase in , then that node is not merged with any communities and remains in its own community. This process proceeds over each node one at a time. Traditionally, the Louvain algorithm continues forward until modularity can no longer be increased. In our study, however, the communities after this first pass only were used. Based on recent evidence, the Louvain algorithm was used to identify unidimensional structures [11] in addition to multidimensional structures.$QQ$

The Louvain algorithm is stochastic in that its node placements depend on the order of the nodes (proceeding from the first to the last). By default, the igraph package (version 2.0.3 [12]) shuffles the node order each and every time the Louvain algorithm is applied. To overcome the stochastic nature of the algorithm, the consensus approach was applied [13]. This approach works by applying the algorithm times (e.g., 10,000) to the same network to identify a “consensus” solution. We applied this approach and used the most common solution across the 10,000 applications. $N$

## S1.4 Missing Data in Embeddings (Derivatives) and Gaussian Noise Jittering

Although missing data was minimal, even a few missing observations in key places could obstruct achieving reliable results. The main issue of missing data in the present approach is in the estimation of the derivatives. In Boker and colleagues [14], bias from missing values and time interval misspecification were evaluated in the time delay embedding (i.e., consecutive windows of time used to estimate the derivatives) and found similar performance in various methods of “doing nothing” (i.e., ignoring the missing data), “row-wise” (i.e., adjust time interval with respect to each embedding, ensuring time intervals are adaptive to missing data), “skipover” (i.e., “skips over” missing data and treats the non-missing points as continuous points in time), and “Kalman smoothing” (i.e., method to impute missing values). We found that the Kalman smoothing method performed best, estimating more individual family networks (40 out of 40) than “doing nothing” (28 out of 40). Specifically, this approach models the underlying temporal dependencies (trend, autocorrelation) to generate estimates for missing observations while preserving the original time scale (see also, Durbin & Koopman, 2001; Gardner et al., 1980). This approach is computationally more intensive than the other methods but typically yields the most accurate imputations by accounting for the stochastic properties of the time series.

Missing data were handled using Kalman smoothing to generate estimates for the missing observations while preserving the original time scales [15,16]. We identified instances of zero-variance derivatives for some variables, such as an individual consistently reporting the same score of “1 = Not at all” for an emotion like “hostile” across all observations in the time period. For variables with zero or near-zero variance, Gaussian noise is generated from a multivariate normal distribution with a near-zero standard deviation (SD = 0.001). The jitter preserves the overall structure but avoids singular covariance matrices during network estimation. This procedure stabilizes matrix inversion without meaningfully altering the underlying temporal structure of the data.

## S1.5 Zero-Variance Derivatives

Zero-variance derivatives were observed primarily in the dataset for negative affective states. For example, a family member within a family triad would demonstrate a zero-variance derivative for a certain negative affective state (e.g., “Hostile”) if they had consistently self-reported the same score (e.g., “1 = Not at all”) for every single observation in the time period. We found that this was somewhat common for participants with negative emotion words (e.g., “Hostile”, “Anger”, “Resentful”). To rectify this, a small amount of Gaussian noise (i.e., “jitter”) was added to zero-variance derivatives to prevent dynamic network estimation failures. For *p* variables that exhibit zero or near-zero variance, Gaussian noise is generated from a multivariate normal distribution with a standard deviation of zero (*SD* = 0.001) for each person—this ensures that orthogonal noise is added. The jitter preserves the overall structure but avoids singular covariance matrices during network estimation. This procedure stabilizes matrix inversion without meaningfully altering the underlying temporal structure of the data. The default jitter magnitude (0.001) is sufficiently small relative to the scale of the EMA variables to avoid influencing substantive network results while ensuring numerical stability during estimation.

## S1.6 Assessing Synchrony of Family Emotions

Level 2 (Family-Level) Synchrony. Definitions of synchrony vary. From a dynamic network framework perspective, we conceptualize synchrony in the current study as the overlap in the rates at which two (or more) variables change together over time [17]. To quantify overlap, Unique Variable Analysis (UVA [18]) was applied to the estimated dynamic networks for each family triad. Using a graph theoretic approach, for each network , we computed the absolute value of the weighted topological overlap (Ω, or wTO [19]) for each pairwise node combination in the network:$W$

$${wTO}_{ij}=\left| \frac{\sum W_{iu}+W_{uj}}{min\{k_{i}k_{j}\}+1-W_{ij}} \right| .$$

Weighted topological overlap values represent the extent to which two variables change together and similarly change with other variables. For each pair of nodes, the wTO represents general synchrony among the variables without directionality (i.e., overall magnitude of synchrony), where smaller values closer to 0.00 indicate less synchrony and greater values indicate greater synchrony. Thus, in this study, family emotional synchrony is operationally defined as the wTO (i.e., synchrony, or overlap) of emotional state nodes over time within family networks. It is computed between each pair of variables with one node for each family member's emotional state (e.g., child-happy with mother-happy, child-happy with dad-happy, child-happy with mother-calm). The overall emotional synchrony “score” for each family system is calculated as the absolute value of the sum of signed pairwise wTO values. When taken together, the sum measures the extent to which the family, as an emotional system, is synchronized; higher synchrony values indicate that the family’s system of affective states change in similar ways over time.

**Level 1 (Momentary-Level) Family Emotional Synchrony**

Momentary family emotional synchrony was operationalized as the degree of similarity in concurrent affect ratings among family members at each EMA prompt. For each of 18 SAS items (9 PA; 9 NA), absolute differences were computed for each dyad within the triad (child–mother, child–father, father–mother). These item-level discrepancy scores were averaged within valence to yield dyad-level discrepancy indices, which were then averaged across the three dyads to obtain family-level momentary discrepancy scores. To facilitate interpretation such that higher values reflected greater synchrony, discrepancy scores were reverse-coded using the formula. Thus, this resulted in momentary PA synchrony and momentary NA synchrony variables ranging from 0 to 4, with higher values indicating greater similarity in family members’ affect at that prompt. When data were partially missing, scores were computed using available item and dyad information (i.e., pairwise deletion within row means).

## S1.7 Multilevel Modeling

Regression and multilevel modeling will be used to assess the extent to which family emotional synchrony predicts adolescent outcomes in daily life. Before estimating the multilevel models, we examined multicollinearity among the overall family emotional synchrony predictors (i.e., positive affect wTO and negative affect wTO). Pearson’s correlations between the overall PA and NA emotional synchrony variables were significantly positive (p < .001), but not perfectly correlated, r = .80. We further examined variance inflation factors (VIFs) using the car package in R [20]. Specifically, we computed VIFs using an ordinary least squares model with the same fixed effects, including both overall emotional synchrony variables (PA and NA) as predictors of overall adolescent outcomes. Various rules of thumb have been proposed in the literature, but VIFs > 10 are generally considered problematic, indicating severe multicollinearity. For the current study, the VIFs for the synchrony variables were moderately high, but not problematic. In linear models predicting adolescent outcomes of stress and well-being (measured at every moment), the multicollinearity values for overall PA emotional synchrony and NA emotional synchrony were VIF = 2.27. The Pearson’s correlation between momentary PA emotional synchrony and NA emotional synchrony was statistically significant, but with a very small effect size, r = .09, p < .001.

Intraclass correlations (ICCs) were calculated for models with momentary outcome variables (stress, and PERMA elements). The ICC quantifies the proportion of variance attributable to mean differences among individuals, thereby indicating the amount of variance that remains to be explained at the within-person level. For intensive longitudinal studies using self-reports, ICCs typically range from .20 to .40; moreover, ICC values > .10 warrant the use of multilevel models [21]. The ICCs in the current study exceed the threshold of > .10, indicating that multilevel models are appropriate for the analyses: stress = .60, Positive emotions = .66, Engagement = .60, Relationships = .75, Meaning = .84, Accomplishment = .75. In this study, certain variables, such as Relationships, Meaning, and Accomplishment, had moderately high ICC values (> .70), indicating greater between-person variance than within-person variance and greater stability over time.

For the second research aim, a series of single-level regressions and two-level linear mixed-effects (random-intercepts only) multilevel models was estimated for each of the adolescent outcome variables of interest. For RQ2a, all predictor variables and outcome variables were at Level 2 (single-level regression). In other words, we assessed whether an adolescent’s overall family emotional synchrony (in PA and NA) was associated with their own overall well-being and mental health (stress, depression, anxiety). The overall emotional synchrony variables were operationalized as the non-signed sum of wTO values, which was then grand-mean centered. We conducted separate models for each element of PERMA, perceived stress, and the DASS-21 facets (depression, anxiety, stress). The equation is as follows:

$${{AdolescentOutcome}_{i}= \beta_{0}+\beta_{1}{(Overall PA Sync)}_{i}+ \beta_{2}{(Overall NA Sync)}_{i}+\varepsilon_{i}}$$

For RQ2b, we estimated a two-level linear mixed-effects model examining whether momentary fluctuations in family emotional synchrony were associated with adolescents’ momentary well-being outcomes. The outcome variables were at Level 1 (mPERMA and momentary stress) with predictor variables of momentary PA and NA emotional synchrony (Level 1) and overall PA and NA emotional synchrony (Level 2). We chose random intercepts models instead of random slopes models because we did not expect any theoretically systematic or substantial change (i.e., random slopes) in stress or well-being due to time over a short timescale of one week of an adolescent’s typical life. Thus, Level 1 in the multilevel models for the current study represents the within-person variances in stress and well-being from moment to moment. The Level 1 predictors were person-mean centered. The predictor variables for overall emotional synchrony were entered as Level 2 predictors, since they indicate that the adolescent came from a family with a certain overall level of emotional synchrony (which differs from that of another adolescent and their family). We conducted separate models for each element of mPERMA and momentary stress. The equation is as follows, where Level 1:

$$MomentaryOutcome = \beta_{0} +\beta_{1}{(Momentary PA Sync)}_{it} +\beta_{2}{(Momentary NA Sync)}_{it} + e_{it}$$

For the Level 1 equation, $\beta_{0}$is the adolescent-specific intercept, $\beta_{1}$and $\beta_{2}$represent the fixed effects of momentary PA and NA synchrony (respectively), and $e_{it}$represents the Level 1 residual error.

Level 2 is as follows:

$$\beta_{0i} = \gamma_{00} + \gamma_{01}{(Overall PA Sync)}_{i} + {\gamma_{02}{(Overall NA Sync)}_{i}} + u_{0i}$$

For the Level 2 equation, the Level 1 slopes are fixed, so $\beta$_1_ = γ_10_ and $\beta$_2_ = γ_20_.

The combined model is:

$MomentaryOutcome = \gamma_{01}+ \gamma_{10}{(Momentary PA Sync)}_{it} + \gamma_{10}{(Momentary NA Sync)}_{it}$ ${+\gamma}_{10}{(Overall PA Sync)}_{it} + \gamma_{10}{(Overall NA Sync)}_{it} + u_{0i} + e_{it}$

At Level 1, momentary adolescent outcomes were modeled as a function of momentary PA synchrony and momentary NA synchrony. At Level 2, the adolescent-specific intercept was modeled as a function of overall family PA synchrony and overall family NAsynchrony, indexed using wTO aggregated across the study period. Random intercepts were included for adolescents to account for the nesting of repeated momentary observations within individuals. The Level 1 synchrony slopes were specified as fixed effects.

# References

1. Jenkins BN, Cross MP, Donaldson CD, Pressman SD, Fortier MA, Kain ZN, et al. The subcomponents of affect scale (SAS): validating a widely used affect scale. Psychol Health. 2023;38(8):1032-1055. doi:10.1080/08870446.2021.2000612

2. Golino H, Christensen AP, Moulder R, Kim S, Boker SM. Modeling latent topics in social media using dynamic exploratory graph analysis: The case of the right-wing and left-wing trolls in the 2016 US elections. Psychometrika. 2022;87(1):156-187. doi:10.1007/s11336-021-09820-y

3. Deboeck PR, Montpetit MA, Bergeman CS, Boker SM. Using derivative estimates to describe intraindividual variability at multiple time scales. Psychol Methods. 2009;14(4):367-386. doi:10.1037/a0016622

4. Friedman J, Hastie T, Tibshirani R. Sparse inverse covariance estimation with the graphical lasso. Biostatistics. 2008;9(3):432-441. doi:10.1093/biostatistics/kxm045

5. Chen J, Chen Z. Extended Bayesian information criteria for model selection with large model spaces. Biometrika. 2008;95(3):759-771. doi:10.1093/biomet/asn034

6. Epskamp S, Fried EI. A tutorial on regularized partial correlation networks. Psychol Methods. 2018;23(4):617-634. doi:10.1037/met0000167

7. Golino HF, Epskamp S. Exploratory graph analysis: A new approach for estimating the number of dimensions in psychological research. PLoS One. 2017;12(6):e0174035. doi:10.1371/journal.pone.0174035

8. Golino H, Shi D, Christensen AP, Garrido LE, Nieto MD, Sadana R, et al. Investigating the performance of exploratory graph analysis and traditional techniques to identify the number of latent factors: A simulation and tutorial. Psychol Methods. 2020;25(3):292-320. doi:10.1037/met0000255

9. Blondel VD, Guillaume JL, Lambiotte R, Lefebvre E. Fast unfolding of communities in large networks. J Stat Mech. 2008;2008(10):P10008. doi:10.1088/1742-5468/2008/10/P10008

10. Jimenez M, Abad FJ, Garcia-Garzon E, Golino H, Christensen AP, Garrido LE. Dimensionality assessment in bifactor structures with multiple general factors: A network psychometrics approach. Psychol Methods. 2025;30(4):770-792. doi:10.1037/met0000590

11. Christensen AP. Unidimensional community detection in psychometric networks: A Monte Carlo simulation, grid search, and comparison. Psychol Methods. 2024. Advance online publication. doi:10.1037/met0000692

12. Csardi G, Nepusz T. The igraph software package for complex network research. InterJournal. 2006;Complex Systems:1695. Available from: https://igraph.org

13. Lancichinetti A, Fortunato S. Consensus clustering in complex networks. Sci Rep. 2012;2:336. doi:10.1038/srep00336

14. Boker SM, Tiberio SS, Moulder RG. Robustness of time delay embedding to sampling interval misspecification. In: van Montfort K, Oud JHL, Voelkle MC, editors. Continuous time modeling in the behavioral and related sciences. Cham: Springer International Publishing; 2018. p. 239-258. doi:10.1007/978-3-319-77219-6_10

15. Durbin J, Koopman SJ. Time series analysis by state space methods. Oxford: Oxford University Press; 2001.

16. Gardner G, Harvey AC, Phillips GDA. Algorithm AS 154: An algorithm for exact maximum likelihood estimation of autoregressive-moving average models by means of Kalman filtering. Appl Stat. 1980;29(3):311-322. doi:10.2307/2346910

17. Moulder RG, Boker SM, Ramseyer F, Tschacher W. Determining synchrony between behavioral time series: An application of surrogate data generation for establishing falsifiable null-hypotheses. Psychol Methods. 2018;23(4):757-773. doi:10.1037/met0000172

18. Christensen AP, Garrido LE, Golino H. Unique variable analysis: A network psychometrics method to detect local dependence. Multivar Behav Res. 2023;58(6):1165-1182. doi:10.1080/00273171.2023.2194606

19. Nowick K, Gernat T, Almaas E, Stubbs L. Differences in human and chimpanzee gene expression patterns define an evolving network of transcription factors in brain. Proc Natl Acad Sci U S A. 2009;106(52):22358-22363. doi:10.1073/pnas.0911376106

20. Fox J, Weisberg S. An R companion to applied regression. 3rd ed. Thousand Oaks (CA): Sage; 2019.

21. Bolger N, Laurenceau JP. Intensive longitudinal methods: An introduction to diary and experience sampling research. New York: Guilford Press; 2013.
